# Assessing factors driving blockchain technology in the performance of the banking sector using the structural equation modelling approach

**DOI:** 10.12688/openreseurope.23147.2

**Published:** 2026-08-31

**Authors:** Pradip Sanatkumar Padhye, Dipak Santram Vakrani, Sushil Kumar Gupta, Jewel Kumar Roy, Neha Parashar

**Affiliations:** 1 Symbiosis International (Deemed University) Symbiosis School of Banking & Finance, Pune, Maharashtra, India; 2 MIT College of Management, MIT Art Design and Technology University, Pune, Maharashtra, India; 3 School of Economics & Commerce, Dr Vishwanath Karad MIT World Peace University, Pune, Maharashtra, India; 4 Doctoral School of Regional and Business Administration Sciences, Szechenyi Istvan University, Győr, Gyor-Moson-Sopron, 9026, Hungary

**Keywords:** Blockchain Technology; Banking Performance; IT Alignment; Technology Adoption; India; SEM analysis

## Abstract

The integration of blockchain technology (BCT) within the banking sector is transforming how organisations achieve operational efficiency and trust. However, the adoption of BCT in the Indian banking sector remains underexplored, particularly regarding organisational alignment and performance. This study explores the critical factors of technology and organisation influencing the adoption of blockchain and its effects on banking performance, with a distinct interest in the moderating effects of IT alignment. Also, to explore whether perceived usefulness and perceived ease of use act as mediators between blockchain technology and banking performance. Using the Technology-Organisation-Environment (TOE) and Technology Acceptance Model (TAM), data were collected from 326 professionals working in prominent Indian banks and FinTech companies. Analysis of the relationships was conducted with Structural Equation Modelling (SEM). The results of this study show that technology readiness significantly influences both perceived usefulness and perceived ease of use, while the regulatory environment significantly affects perceived usefulness. Furthermore, IT alignment significantly moderates the relationship between blockchain technology and business performance, and perceived usefulness serves as a significant mediator, whereas perceived ease of use does not significantly mediate this relationship. The study provides a theoretical contribution by incorporating the TAM and TOE models in a banking context and serves to inform practitioners by providing insights into blockchain adoption strategies in Indian financial institutions.

## 1. Introduction

Blockchain technology, which is a protected, circulated register, has become a ground-breaking invention that can improve decentralisation, security, and transparency. Blockchain makes it harder to change recorded information by ensuring data immutability using cryptographic processes. ^1^ The potential of blockchain to enhance supply chains, reduce fraud, and boost accountability in financial transactions is frequently lauded. ^2^ However, little attention is paid to the socio-technical and legal issues that affect its uptake in these areas. From scepticism to broad adoption, blockchain has gained popularity, especially in nations like the US and Japan. ^3^ For instance, Japan accepted Bitcoin as legal currency in 2017, ^4^ while Estonia included it in its e-governance system in 2014. The use of Bitcoin as legal cash by Cooper and Kruglikova ^5^ further demonstrates the potential of blockchain technology. These cases, however, frequently ignore the difficulties faced by underdeveloped nations, such as inadequate infrastructure and unclear regulations. The banking industry could benefit from blockchain, according to a large portion of the research now in publication. ^6^ Asset change, liquidity storage, and the use of economies of scale to deliver financial services are the main functions of banks. ^7^ Banks employ a managerial middleman to streamline the loading of resources for future use and funding bases, with distinct procedures for all parties involved. As a result, all banking operations are controlled as a collection of consolidated records for payments, adaptation, and services concerning the assets of investors. The growth of blockchain technology might help the economic system move from centralised to decentralised management. A centralised record of communications that is readily visible to all users forms the basis of the technology known as blockchain, allowing trust to grow in dangerous circumstances without a requirement for intermediaries. The agreed and established state of the digital chain is included in the ledger, together with an unchangeable record of all prior transactions.

As the banking and financial sector shifted to branchless banking, digital value exchange, and payments via mobile devices, the technology known as blockchain emerged, predicting a worldwide financial system collapse. The integration of blockchain-enabled technologies with conventional financial systems will improve services for both banked and unbanked customers worldwide, according to Wang et al. ^8^ Blockchain-based knowledge can help economic services companies with a variety of tasks, including trading in Bitcoin, bond operations, swaps of currencies, check issuing, improved KYC, improved settlement, loan approval, money transfers, intelligent agreements, trade financing, and other processes. Furthermore, to adapt innovation to the issues at hand, blockchain can be used in combination with other technologies like identity management, encoding, and business rules. ^9^ Research communities are debating different frameworks, blockchain concepts, and applications. Monetary asset settlement, services associated with commerce, and market forecasts are just a few of the many banking applications that are currently utilizing blockchain technology. ^10^ In the future, blockchain is anticipated to be crucial for sustained global economic expansion, which will benefit consumers and the public alike. ^11^

### 1.1 Research Contribution and Problem Statement

The rapid advancement of digital technologies has made a paradigm change in the monetary services segment, in which the blockchain is evolving as a transformational force, especially in the investment sector. Despite its ability to increase transparency, safety, and operational efficiency, ^12^ adopting blockchain technology in banking is inconsistent and fragmented. This uneven adoption is often attributed to a complex set of technical, directorial, and environmental factors. In the context of Indian banking, unique socio-technical and regulatory challenges such as strict compliance requirements, legacy IT systems, and data privacy concerns further complicate the adoption process. While many banks accept the strategic importance of blockchain in reducing fraud, improving transaction speed, and reducing costs, ^13^ a clear understanding of the major determinants affecting its implementation is still lacking. There is a gap in empirical research that examines these adoption factors in the Indian banking environment, which is situated in separate legal, cultural, and technological contexts from those of Western economies. Although studies such as Garg et al. ^14^ and Jena ^15^ have examined blockchain adoption and performance in Indian banking and financial institutions, important research gaps remain. Garg et al. ^14^ focused on the relationship between blockchain capabilities, competitive advantage, and organisational performance, while Jena ^15^ investigated blockchain adoption using an extended UTAUT framework. However, neither study examined the role of IT Alignment as a moderating factor influencing blockchain-related outcomes. Furthermore, previous studies have not compared the relative influence of Perceived Usefulness (PU) and Performance Expectancy (PE) as competing predictors within the same research model. This study addresses these gaps by examining the moderating role of IT Alignment and comparing the effects of PU and PE in explaining blockchain adoption and performance outcomes in the Indian banking context.

In addition, sector-specific obstacles that the regulator faces in the integration of blockchain include the resistance to uncertainty, data secrecy concerns, and resistance to change in traditional banking infrastructure. ^16^ Existing studies have mainly used qualitative or descriptive methods to detect these issues, which limits the generality of conclusions. Therefore, this study adopts SEM approach to an empirical assessment of driving factors, such as alleged utility, ease of integration, ease of integration, belief, and regulatory support, facing blockchain adoption in banking. By focusing on the Indian banking sector, this study provides contextual insights that can be used by policymakers and financial institutions directing the digital transformation drive. By doing this, the purpose of the research is to provide a strong, data-managed understanding of how these interconnected variables affect the decisions of adoption, which provides insight to policymakers, technology providers, and financial institutions trying for digital changes. ^17^

### 1.2 Research Question and Objectives

The analysis’s primary goal is

• To ascertain how the factors of blockchain technology affect banking performance.
• To look into how IT Alignment moderates the association between blockchain technology and banking performance.
• To determine the mediating role of perceived usefulness and perceived ease of use between blockchain technology and banking performance.

Based on the aforementioned goal, the study questions are as follows:

**RQ1:** How does blockchain technology (security & efficiency, trialability, reduction in the cost, and regulatory compliance) affect banking performance?

**RQ2:** Does IT Alignment moderate the association between blockchain technology and banking performance?

## 2. Literature Review

The integration of blockchain technologies in the banking sector has gained momentum, with research focusing on factors like security & efficiency, trialability, reduction in the cost, and regulatory compliance that drive its adoption and influence performance. Studies using Structural Equation Modelling (SEM) highlight how these elements, along with innovation capacity, shape organisational outcomes. The majority of these studies utilise well-established theoretical frameworks such as the Technology Acceptance Model (TAM), Unified Theory of Acceptance and Use of Technology (UTAUT), Technology–Organisation–Environment (TOE) framework, and the Resource-Based View (RBV). This section reviews and synthesises the most relevant and recent works that explore blockchain adoption within the banking and financial services sectors. Table 1 outlines the major studies supporting this analysis.

**Table 1.** Evaluation of Related Works.

Author(s)	Theory/Model Used	Blockchain Adoption/Use	PU	PEU	Organizational Factors	Method (SEM/ANN/PLS)	Sample
Al Mamun et al. ^18^	TAM	✓(IBT)	✓	✓	X	SEM	380 banking workers
Jena ^15^	UTAUT	✓	✓	X	X	Survey (SEM implied)	Bankers & FinTech pros
Chengyue et al. ^19^	Extended TAM	✓	✓	✓	X	SEM	287 experts (multi-sector)
Bag et al. ^20^	TOE & RBV	✓(BCA, MAP)	X	X	✓(TMS, Preparedness)	ANN & CB-SEM	311 SMEs (South Africa)
Legesse et al. ^21^	TAM + TOE	✓	✓	✓	X	PLS-SEM + ANN	178 NQI experts
Kabir et al. ^22^	UTAUT + Open Innovation	✓(BINTU)	✓	✗	X	SmartPLS-SEM	IPDC staff
Garg et al. ^14^	RBV & Mediation Framework	✓	X	X	✓(BCC, Efficiency, CA)	SEM + PROCESS macro	289 finance experts
Sciarelli et al. ^23^	Extended TAM	✓	✓	✗	X	PLS-SEM	108 Italian SMEs

Summary of key empirical studies on blockchain adoption in banking and financial services, detailing author(s), theoretical framework, constructs examined, methodology, and sample characteristics.

### 2.1 Related Works on Blockchain Adoption in Banking

**2.1.1 Technology Acceptance Model (TAM)-Based Studies**

The Technology Acceptance Model (TAM) asserts that Perceived Usefulness (PU) and Perceived Ease of Use (PEU) are two critical determinants of a user’s intention to adopt a new technology. Al Mamun et al. ^18^ addressed how TAM constructs impact blockchain adoption in the banking sector in Bangladesh, including additional constructs such as Perceived Compliance and Perceived Environmental Variables. They used Structural Equation Modelling (SEM) to examine data collected from 380 banking professionals. The results revealed that both PU and PEU have a significant effect on a banking professional’s Intent to Use (ITU) blockchain technology, and ITU consequently affects sustainability performance, indicating ITU is a mediator of the relationship. Chengyue et al. ^19^ also investigated blockchain adoption using a TAM-based framework that included Risk, Trust, and Expertise as additional components of their behavioural intention to adopt blockchain technology in cross-industry systems (CIS). They applied AMOS-SEM analysis of data pulled from 287 professionals, and results suggested that Perceived Benefits, Convenience of Use, Innovation, and Trust were positively related to adoption of blockchain technology, while Risk had no significant influence. Sciarelli et al. ^23^ built upon TAM and introduced two additional constructs: Reduced Cost and Efficiency & Security, and examined blockchain adoption again in Italy (108 data points collected, PLS-SEM analysis). The findings demonstrated that Efficiency and Security significantly influence Perceived Usefulness, which strongly predicts Intent to Use blockchain technology in business processes.

**2.1.2 UTAUT and Extended UTAUT-Based Studies**

The Unified Theory of Acceptance and Use of Technology (UTAUT) framework consists of constructs appropriate to the context, such as Performance Expectancy, Effort Expectancy, Social Influence, and Facilitating Conditions, making it especially useful for understanding technology adoption in organisations. Jena ^15^ applied an extended UTAUT model that examined blockchain adoption in Indian financial institutions as well as FinTech organisations. The study found that Initial Trust, Performance Expectancy, and Favourable Conditions were the largest predictors of adoption intention. Initial Confidence, particularly, acted as a moderator, demonstrating how trust serves as an essential enabler in blockchain adoption. Similar to Jena, ^15^ Kabir et al. ^22^ even took the UTAUT model a step further, combining it with an open innovation viewpoint to study blockchain utilisation in supply chain finance, specifically at IPDC Finance in Bangladesh. They found, using SmartPLS-SEM, that Facilitating Conditions (FCON) significantly affected Behavioural Intention to Use (BINTU), which mediated the effects of other constructs like Performance Expectancy and Social Influence. They also found that Social Influence did not significantly affect Behavioural Intention, suggesting that both individual and organisational contexts may influence its effect. Pasquale Sarnacchiaro et al. ^24^ studied blockchain adoption using the UTAUT model with additional factors such as trust and security. The study aimed to understand the factors influencing users’ intention to adopt blockchain technology in tourism. The findings showed that trust and security are important factors that encourage blockchain adoption. Although the study focused on tourism, it highlights the importance of these factors in blockchain adoption, which is also relevant to the banking sector.

**2.1.3 TOE Framework-Based Studies**

The Technology–Organisation–Environment (TOE) framework is a way to categorise the dimensions and factors influencing technology adoption (i.e., technological, organisational, and environmental). Legesse et al. ^21^ leveraged the TAM and TOE frameworks to evaluate blockchain adoption in Ethiopia’s National Quality Infrastructure (NQI). Their study featured both internal (e.g., PU, PEU, ITU) and external (Technology Integration, Government Support, Policy) factors. Using PLS-SEM and ANN, they concluded that Technology Compatibility, Perceived Utility, and Support from Senior Management positively affected blockchain adoption decisions, thus illustrating the importance of both internal preparedness and external compatibility. Bag et al. ^20^ also adopted the TOE framework (along with RBV) to assess blockchain adoption among South African SMEs. The results from their CB-SEM and ANN methods indicated that Relative Advantage, Organisational Readiness, Support of Top Management, and External Pressures (i.e., regulations) positively affected Blockchain Capability Adoption (BCA) and Managerial Adoption Performance (MAP). They established that Complexity harmed adoption and emphasised the need for blockchain to be simplified for implementation.

**2.1.4 Resource-Based View (RBV)-Based Studies**

The Resource-Based View (RBV) theory examines how distinct resources and capabilities of an organization to a competitive advantage and improved performance. Garg et al. ^14^ applied RBV to create a model that examined a link between Blockchain Capabilities (BCC), Competitive Advantage (CA), and Organisational Performance (OP). Using 289 data points from experts in Banking and Financial Services, their SEM analysis using the PROCESS macro demonstrated BCC had a significant impact on CA, resulting in CA partially mediating BCC on OP. This presentation identifies a strategic role of blockchain capabilities in driving competitive advantage and organisational performance. Bag et al. ^20^ also emphasised the role of internal resources such as Top Management Support, Organisational Preparedness, and Technological Compatibility in their dual application of the TOE and RBV to further enable realization of blockchain capabilities. These authors validated the stability of these predictors through the use of ANN and CB-SEM techniques.

### 2.2 Research Gap and Motivation for the Study

The review of existing literature reveals that although substantial progress has been made in identifying the drivers of blockchain adoption in various sectors, including banking and SMEs, there remains a gap in fully understanding the combined influence of technological, organizational, and environmental factors, particularly in emerging economies and highly regulated sectors most studies assess either individual-level constructs (e.g., PU, PEU, trust) or organizational resources (e.g., TMS, ORD) while few combine those lower and higher level constructs into overarching models without much theory implications. Limited studies explore multiple theoretical lenses either cumulatively (e.g., TAM, UTAUT, TOE) when explaining both the blockchain adoption behaviour and consequential performance impacts in banking. This study will address these research gaps through the development of an integrative research model, building upon the most relevant constructs from TAM, UTAUT, TOE, and RBV to further investigate the following: blockchain adoption and the impacts on organisational performance in banking. By utilising advanced methods of analytical techniques (e.g., SEM and potentially ANN as well), this study plans to offer practical and theoretical contributions to enhance both theoretical understanding and provide suggestions assisting financial institutions with blockchain efforts.

Although previous studies have examined blockchain adoption in the Indian banking and financial services sector, important research gaps remain. For example, Jena ^15^ investigated blockchain adoption in Indian financial institutions using an extended UTAUT model, while Garg et al. ^14^ examined blockchain capabilities and organisational performance using an RBV-based SEM approach. However, neither study examined IT Alignment as a moderating variable influencing blockchain adoption and organisational performance. Furthermore, previous studies have not compared the relative influence of Perceived Usefulness (PU) and Performance Expectancy (PE) as competing predictors within the same research model. Therefore, the present study addresses these gaps by incorporating IT Alignment as a moderator and examining the comparative effects of PU and PE on blockchain adoption and organisational performance in the Indian banking sector.

### 2.3 Theoretical Underpinnings

**2.3.1 Technology-Organization-Environment**

The TOE framework is an empirical framework that clarifies how technology is adopted within businesses and how the technical, corporate, and ecological settings all affect the process of embracing and carrying out technical innovations. The model was released in 1990 by Baker. ^25^ According to Awa et al., ^26^ the model of TOE is intended for an organisational-level study. Rather than concentrating on the detailed behaviours of persons within the organisation, the framework emphasises more advanced attributes, including the technological, environmental, and organisational contexts. Behavioural models like the model of technology acceptance, the theory of action that is rational action, and the concept of planned behavior should be used to comprehend technology acceptance at the person level. Although this division between individual-level and organization-level theory is widely acknowledged, it also makes it challenging to look into the higher-level characteristics. Since data can only be gathered from members of the target group, it is inherently skewed by the opinions of those individuals. When individual perception is considered, Li ^27^ has shown that behavioral paradigms and the TOE framework are roughly equivalent. The structure of the TOE is “too generic” and provides a great degree of freedom to vary elements and metrics; therefore, there is little need to alter the theory itself, according to Zhu and Kraemer. ^28^ This is the cause for the lack of progress. The fact that the theory does not provide competitive reasons and fits “too well” with different theories of technology adoption is another significant factor, according to Baker et al. ^29^ As a result, there is not much pressure to change the framework.

**2.3.2 Technology Acceptance Model**

The TAM is a widely recognized and extensively employed empirical framework to comprehend how people accept and use technology. ^30^ TAM posits that user attitudes toward technology, including perceived utility and ease of use, noteworthy impact their intent to adopt and use the technology. Additionally, social influence, such as the opinions of peers or experts, plays a critical role in influential individuals’ perceptions and acceptance decisions.

Numerous studies have established the applicability and relevance of TAM in numerous research contexts. ^31, 32^ In the research, ^32^ the authors delve into the aspects impacting blockchain acceptance among Italian companies. They employ an extended TAM model to develop a conceptual framework and test hypotheses using PLS-SEM on a sample of 250 firms. The findings shed light on the determinants of blockchain acceptance and reveal the moderating role of firm size. This research offers valuable insights into the acceptance of blockchain knowledge in the Italian context. Another noteworthy paper, ^31^ investigated the features impacting the intent of tourism and hospitality SMEs to adopt cryptocurrency payments. The study integrated strategic orientation, social influence, and owner/manager characteristics into the technology acceptance model. By examining their impact on perceived utility and ease of use, the paper provides an empirical contribution to understanding technology adoption in SMEs within the hospitality sector. The authors extended the TAM to assess key elements such as perceived worth, ease of use, and innovativeness. This research contributes to our consideration of consumer behavior and their attitudes towards an emerging financial technology in the cases considered.

### 2.4 Hypothesis Development

**Figure 1.**
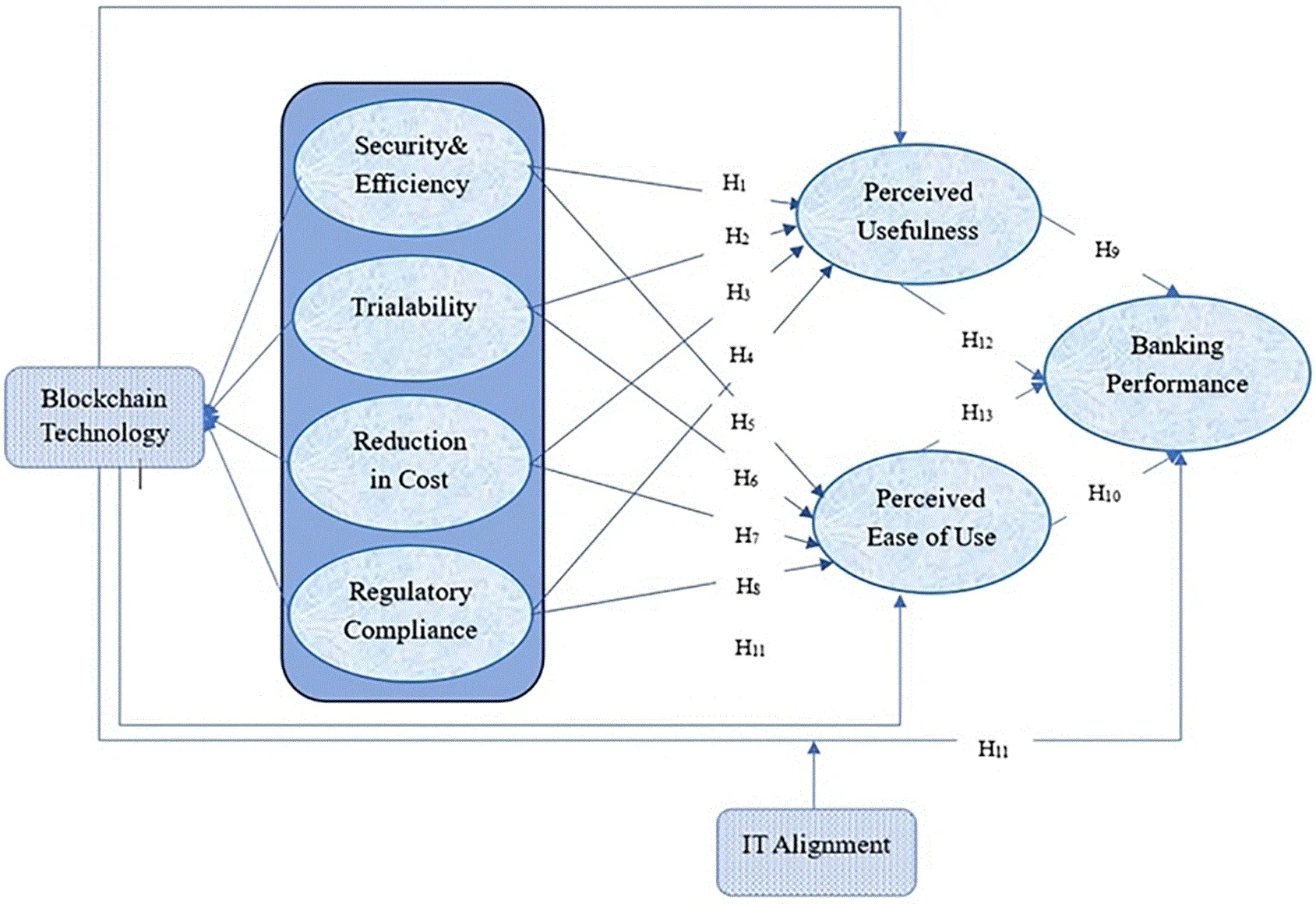
Hypothesis Framework. Conceptual model illustrating the relationships between blockchain technology determinants (Security & Efficiency, Trialability, Reduction in Cost, Regulatory Compliance), mediating variables (Perceived Usefulness, Perceived Ease of Use), moderating variable (IT Alignment), and Banking Performance. The framework integrates TOE and TAM theoretical perspectives.

This study examines a novel outline for Blockchain adoption that integrates the TOE and utilises it to recognise the essentials that affect the acceptance of Blockchain by Indian banks and fintech companies. Additionally, this encompasses technology readiness elements, such as trialability, organisational readiness, security considerations, such as cost savings, and environmental readiness factors, such as regulatory compliance. Additionally, by using TAM to evaluate individual-level views of the utility and use of blockchain, this study uses an integrated theoretical approach. This combined use of TAM and TOE benefits both researchers and practitioners by integrating micro- and macro-perspectives and offering insightful information on the aspects influencing blockchain acceptance in the Indian setting. In this division, establish a speculative research standard that forms the foundation for examining and testing the developed hypotheses. Figure 1 visually represents this model, providing a clear illustration of the relationships and variables under investigation. Subsequently, this study presents the hypotheses concerning the specific aspects addressed in the subsequent sub-sections.

**2.4.1 Influential Factors to Blockchain Adoption**

The adoption of blockchain in banks and fintech companies is influenced by several important aspects.

**Security & Efficiency**

Transactional data and private information are safeguarded during transmission via security. Because of its safe database and safeguarding privacy architecture, blockchain technology offers robust information security ^33^ and enables users to conduct transactions without disclosing their identities. Security risks have been found to have a major impact on the adoption of technology in earlier studies. ^34^ Consequently, this study put forth the following hypothesis:

H1: The Security & Efficiency of blockchain technology influence perceived usefulness.

H5: Security & Efficiency of blockchain technology influences perceived ease of use.

**Trialability**

The capacity of an invention to go through limited testing before full implementation is known as trialability. ^35^ The possibility of an invention being successfully adopted is increased when people and organizations are allowed to test it out before implementing it, claim Attaran and Woods. ^36^ According to research by Ramdani et al., ^37^ trialability is a crucial component in the adoption of business applications. As evidenced by their study on the receiving of e-commerce, trialability also has a substantial influence on the implementation of a skill. Before deploying Blockchain SCs, a testing period is crucial, according to another study. As companies move from conventional contracts to blockchain-based SCs, it will be essential to analyse these new commercial systems and technology to build user confidence. As companies move away from conventional structures, this shift will occur. Trials are a useful technique for lowering the possibility of mistakes and issues. The importance of trialability in embracing technological innovations has been demonstrated in previous studies. It follows that trialability will affect the software industry’s acceptance of Blockchain technology. Thus, the hypothesis of this investigation was that:

H2: Trialability of blockchain technology affects perceived usefulness.

H6: Trialability of blockchain technology affects perceived ease of use.

**Reduction in Cost**

“Cost” describes how much money may be saved by implementing the Blockchain system. Blockchain can upend the finance sector and offer affordable solutions by lowering processing costs in banks. ^38^ In line with the goals of management of supply chains and to decrease expenses, it is also anticipated to provide advantages for risk mitigation and expense reduction. ^39^ Thus, this research proposed the following hypothesis:

H3: Reduction in the Cost of blockchain technology has a direct impact on perceived usefulness.

H7: Reduction in the Cost of blockchain technology has a direct impact on perceived ease of use.

**Regulatory Compliance**

In order to monitor technology service providers and consumers and make sure they fulfill their responsibilities and stay out of trouble, the government establishes rules and regulations, often known as government support. ^40^ In order to decriminalize and implement new skills within a nation’s legal agenda, e-commerce and customer service monitoring depend on laws and regulations. To guarantee that all processes are carried out effectively and equitably, these regulations are essential. This also holds regarding consumer perceptions of cryptocurrencies and blockchain technologies. Regulation is required to reduce or eliminate any misunderstanding that may arise and influence customers’ desire to use the technology safely and trustingly. However, further obstacles to the worldwide adoption of cryptocurrencies include insufficient legal frameworks. ^41^ Consequently, this study hypothesized that:

H4: Regulatory Compliance of blockchain technology affects perceived usefulness.

H8: Regulatory Compliance of blockchain technology touches perceived ease of use.

**2.4.2 Perceived Usefulness**

A person’s adoption of technology is shown to be noteworthy inclined by their perception of its utility (PU). The individual views of users on the particular merit of implementing a technology or system that has the potential to improve or worsen users’ work performance are known as PU. ^42^ People often look at new technology to see whether it will improve their effectiveness at work or in other activities. This research aids in shaping how people view technology in terms of improving performance. If and only if perception and experience are not at odds, they will keep using the program. The perceived utility and the intention of behavior to utilize technologies were shown to be significantly correlated by researchers in the cases of internet purchasing goods, electronic government instruction, internet banking, mobile banking, and hotel tablet apps. ^43^ Nuryyev et al. ^31^ corroborate the favourable association between the desire to implement BCT and perceived usefulness. Consequently, one hypothesizes the following:

H9: Perceived usefulness has a noteworthy effect on Banking Performance.

**2.4.3 Perceived Ease of Use**

PEU has been shown in several studies to have a favourable impact on users’ intentions to embrace and use emerging technologies. ^31^^,^^44^ Notably, the results show that people view the technology of blockchain as more beneficial the easier it is to use. ^45^ It is essential to understand how PEU affects perceived utility to understand the acceptance of user behaviour, especially when it comes to the adoption of emerging innovations like blockchain. ^46^ Analyzing this connection offers important insights into the decision-making and adoption habits of users. This makes it easier to pinpoint the elements that influence consumers’ opinions about how simple and helpful a blockchain technology is, which helps to clarify adoption trends and any roadblocks. Based on the results of earlier research and the justification offered, the following hypotheses were proposed:

H10: Perceived ease of use has a positive effect on Banking Performance.

**2.4.4 IT Alignment as Moderator**

Continuous alignment is seen to exist between strategic goals, measures, and achievements. ^47^ The overall success of the company is influenced by a number of company-related organisational disciplines, including strategy, managerial information systems, organisational conduct, and manufacturing strategy, all of which are fundamentally influenced by IT alignment. ^48^ Alignment with blockchain means integrating this distributed database with conventional basic financial systems to provide speed, security, and transaction openness. Banks, whether public or private, would have the chance to update their IT infrastructure to encounter the unique tests posed by decentralised architectures, the legal perspective, and the requirement for real-time data processing, due to blockchain. Consequently, one hypothesis is the following:

H11: IT Alignment moderates the relationship between Blockchain Technology and Banking Performance.

**2.4.5 PU and PEU as a Mediator**

The role of Perceived Usefulness (PU) and Perceived Ease of Use (PEU) as mediating variables has been extensively supported in technology adoption literature, especially within the Technology Acceptance Model (TAM) framework.

This hypothesis proposes that blockchain technology affects banking performance through Perceived Usefulness (PU). Specifically, the more effectively that blockchain technology improves banking operations, including improved transparency, security, and speed of transaction, the greater the banking staff and management perceive the technology to be useful. When banking employees believe blockchain is useful, their decision to adopt and utilize it is augmented, resulting in improved banking performance including operational efficiencies, customer satisfaction, and improved profits. PU serves as an important mechanism linking the implementation of blockchain technology to actual performance improvements in banking institutions. Hypothesis is thus framed using the above discussion:

H12: PU has a mediating effect between Blockchain Technology and Banking Performance.

This hypothesis proposes that Perceived Ease of Use (PEU) serves as a mediator between blockchain technology and banking performance. Even with the possibilities for blockchain technology to provide benefits, the complexity or difficulty in using the technology may serve as a barrier to actual adoption. When users view the blockchain technology as easy to use or as easy to integrate into existing workflows, they are more inclined to adopt it fully, and this enhances the overall performance of the bank. As such, ease of use of the technology leads to implementation that is more successful and user acceptance that is enhanced, which results in more efficient processes, fewer mistakes, and faster completion times, all of which collectively improve banking performance. Thus, PEU is an important factor to consider in a case of blockchain technology adoption to improve bank performance. Assumptions are made below:

H13: PEU has a mediating effect between Blockchain Technology and Banking Performance.

## 3. Research Methodology

### 3.1 Data Gathering: Instrument and Process

The data collection instrument used in this empirical study was a 5-point Likert-scale questionnaire with closed-response questions. It was adapted from validated measurement scales used in similar research to ensure appropriate construct coverage and benefit from the established reliability and validity. The questionnaire underwent peer review, pilot testing, and necessary adjustments to enhance its clarity and eliminate redundancy.

Purposive sampling, a non-probability sampling method, was performed in this research. ^49^ The self-administered primary data collection involved individuals (general managers, heads of departments, and operational employees) with varying levels of firms, diverse educational backgrounds, and work experience from public sector banks, private banks, cooperative banks, and FinTech firms located in Mumbai, Bangalore, Delhi NCR, and Hyderabad. Participants were chosen based on their understanding of blockchain technology, with additional information. A structured online questionnaire was used to capture responses on blockchain technology adoption.

A total of 402 survey questionnaires were initially obtained from the target population, and 76 incomplete replies were excluded from data analysis. Thus, the final sample size for later analysis was 326.

### 3.2 Measurement of Constructs

The research model consists of eight factors ( Table 2): Security and Efficiency (SE), Trialability (TY), Reduction in Cost (RC), Regulatory Compliance (RE), Perceived Usefulness (PU), Perceived Ease of Use (PE), IT Alignment (IA), and Banking Performance (BP). To ensure content validity, all measurement items were modified from scales earlier validated in other studies. Each item was measured on a five-point Likert scale ranging from ‘very strongly disagree’ to ‘very strongly agree’. By leveraging established measurement instruments and modifying them appropriately for the current study context, we aimed to develop a questionnaire that not only captured the key constructs but also benefited from prior validation, strengthening the validity and reliability of the results.

**Table 2.** Constructs of measures.

Constructs	Items		Sources
Security and Efficiency (SE)	SE1	Blockchain technologies will improve security.	^33, 34^
	SE2	Blockchain-based technologies will boost the system’s integrity.	
	SE3	Blockchain technologies will be useful for monitoring corporate transactions in real time.	
Trialability (TY)	TY1	Blockchain technologies will produce an audit trail that cannot be altered.	^35, 36^
	TY2	Blockchain-based technologies will improve the robustness of the system.	
	TY3	Blockchain technologies will make things more resilient.	
Reduction in Cost (RC)	RC1	Blockchain-based technologies will lower the cost of transactions.	^38, 39^
	RC2	Blockchain technologies will do away with middlemen.	
	RC3	The use of blockchain technology will reduce operating expenses.	
Regulatory Compliance (RE)	RE1	Blockchain technologies will make company operations more efficient.	^40, 41^
	RE2	Blockchain-based technologies will guarantee unchangeable corporate regulations.	
	RE3	The use of blockchain technology will stop financial fraud.	
Perceived Usefulness (PU)	PU1	I believe Blockchain Technology improves the bank’s performance.	^42, 43^
	PU2	Blockchain technology has the potential to improve bank work quality.	
	PU3	Using this technology would increase the effectiveness of the banks.	
Perceived Ease of Use (PE)	PE1	Blockchain technologies are well-defined and reasonable.	
	PE2	Using blockchain technology does not require a lot of mental strength.	
	PE3	Blockchain technology is easy to use.	
IT Alignment (IA)	IA1	Our bank’s IT strategy is aligned with its overall business goals.	^47, 48^
	IA2	IT initiatives are integrated into our bank’s operational and innovation strategies.	
	IA3	Our IT infrastructure supports current and future banking requirements.	
Banking performance (BP)	BP1	Our bank has shown improved financial performance in the last 3 years.	^34, 36^
	BP2	Our bank has become more competitive in the market due to digital initiatives.	
	BP3	The bank is able to introduce innovative financial products faster than before.	
	BP4	The overall service quality of the bank has improved through digital transformation.	

Survey items for each construct with corresponding sources adapted from validated measurement scales.

**3.2.1 Second-Order Construct Specification**

In this study, Blockchain Technology (BCT) is conceptualized as a second-order reflective construct represented by four first-order dimensions: Security and Efficiency (SE), Trialability (TY), Reduction in Cost (RC), and Regulatory Compliance (RE). Each first-order dimension was measured using multiple reflective indicators adapted from prior studies. The second-order construct was operationalized using these four validated dimensions, and therefore BCT does not have separate measurement items of its own. The structural relationships involving Blockchain Technology were estimated using the second-order latent construct rather than the individual dimensions.

### 3.3 Demographic Profile of Respondents

This study included 326 participants in the demographic distribution analysis **.** The majority of the sample suspects are male, constituting 80.7% of the sample. Most applicants aged 31–50 years hold Bachelor’s degrees (57.7%); Master’s degree holders follow in rank among the highest education levels represented among respondents. The respondents were somewhat evenly split among public, private, cooperative banks, and Fin-Tech firms. Most of the respondents are general managers, accounting for 46.6% of the general population, and 61.3% have over ten years of experience. The description suggests that it is a highly experienced and well-educated group across diverse banking institutions.

**Figure 2.**
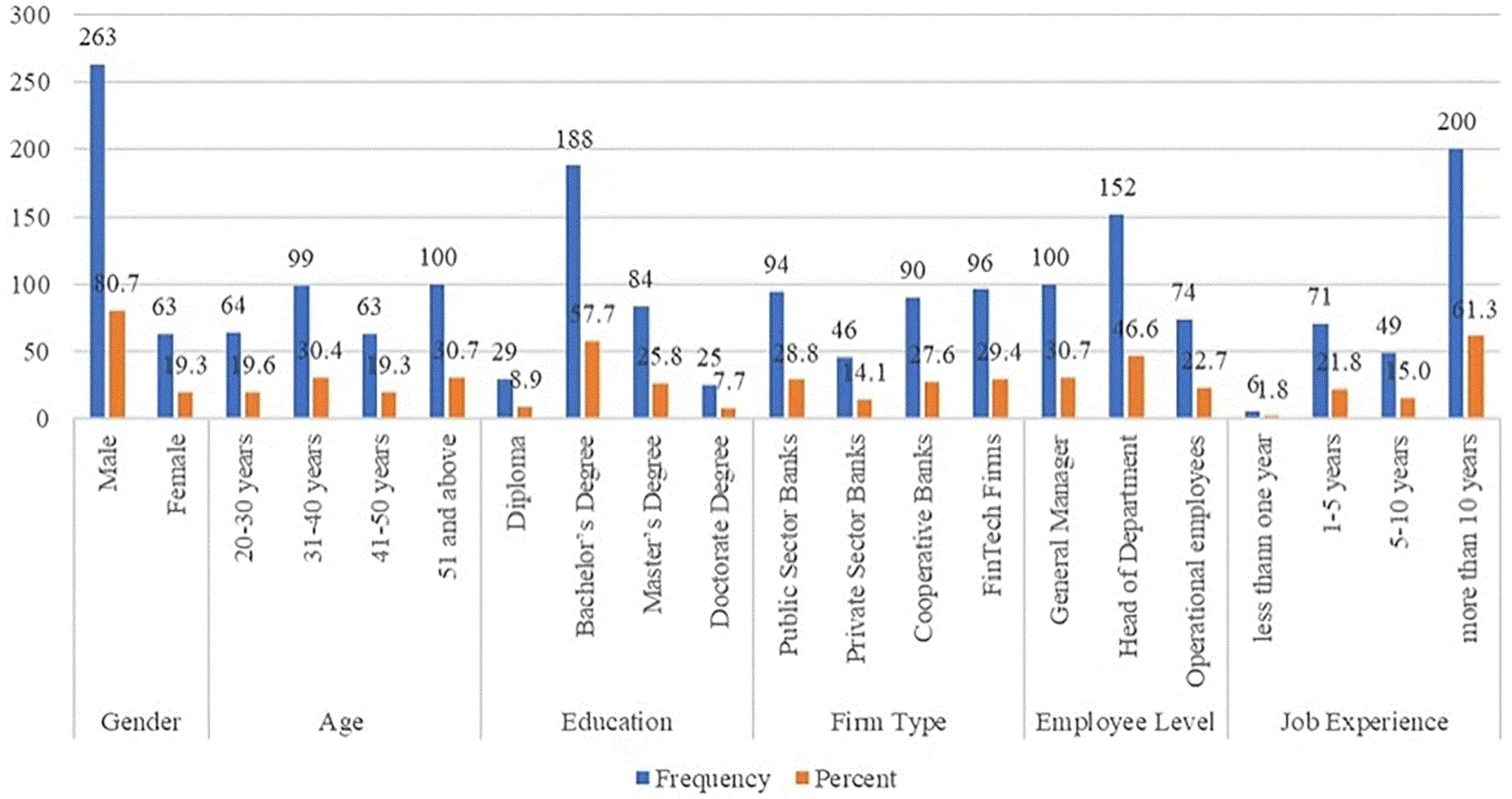
Demographic Profile of Respondents. Distribution of 326 survey participants by (a) gender, (b) age group, (c) education level, (d) organization type, (e) job position, and (f ) years of experience.

Figure 2 presents the demographic profile of the 326 survey respondents, revealing that the sample predominantly comprises male participants (80.7%) aged 31–50 years, with the majority holding Bachelor’s degrees (57.7%) followed by Master’s degrees, employed across public, private, cooperative banks, and FinTech firms, with general managers representing 46.6% of the sample and 61.3% possessing over ten years of professional experience, indicating a highly experienced and well-educated respondent base across diverse banking institutions.

## 4. Data Analysis and Results

Several statistical methods and techniques that have been verified were used to scrutinise the primary data that was assembled. Confirmation of internal consistency and construct reliability was among the crucial phases and actions necessary for the creation of a measuring scale. The factor analysis that was performed on the gathered data proceeded next. Additionally, CFA was employed to guarantee the measuring scale’s validity. The analysis’s findings are described in the subsections that follow. AMOS 26.0 was utilised for CFA in the present research, and SPSS 20 was used for EFA.

### 4.1 Common Method Bias (CMB)

To address CMB in SEM, the VIF was assessed before testing the structural model to identify constructs with high correlation. The results indicated that the maximum VIF value was 2.555, which is lower than the cut-off of 5.00, and is even less than the more conservative value suggested by Kock & Lynn, ^50^ which is 3.3. Furthermore, Harman’s single-factor analysis was conducted. The outcomes exposed that a single factor accounted for only 35.2% of the total variance in the analysis, which is lower than the required cut-off of 50%. ^51^ This shows that common technique bias will probably not be an important issue in the study.

### 4.2 Measurement Model Analysis

The standard of an instrument may be determined by its dependability. ^52^ To guarantee scale validity, a tool’s dependability is also essential. The degree to which test results are stable and reliable is known as an instrument’s statistical dependability. Therefore, maintaining the device’s dependability is essential to producing a trustworthy instrument. Although there are several methods for assessing an instrument’s dependability, internal stability, and consistency are easy to calculate and frequently prove effective in field research assignments. The scale indicates the extent to which a set of items or assertions has been standardized, and this is the internal texture score ( Table 3).

**Table 3.** Measurement Model.

Constructs	Items		SFL	CA	CR	AVE	MSV
Security and Efficiency (SE)	SE1	Blockchain technologies will improve security.	0.821	0.778	0.78	0.542	0.133
	SE2	Blockchain-based technologies will boost the system’s integrity.	0.824				
	SE3	Blockchain technologies will be useful for monitoring corporate transactions in real time.	0.779				
Trialability (TY)	TY1	Blockchain technologies will produce an audit trail that cannot be altered.	0.805	0.885	0.886	0.722	0.339
	TY2	Blockchain-based technologies will improve the robustness of the system.	0.886				
	TY3	Blockchain technologies will make things more resilient.	0.848				
Reduction in Cost (RC)	RC1	Blockchain-based technologies will lower the cost of transactions.	0.862	0.862	0.863	0.679	0.012
	RC2	Blockchain technologies will do away with middlemen.	0.889				
	RC3	The use of blockchain technology will reduce operating expenses.	0.896				
Regulatory Compliance (RE)	RE1	Blockchain technologies will make company operations more efficient.	0.902	0.904	0.905	0.762	0.065
	RE2	Blockchain-based technologies will guarantee unchangeable corporate regulations.	0.914				
	RE3	The use of blockchain technology will stop financial fraud.	0.905				
Perceived Usefulness (PU)	PU1	I believe Blockchain Technology improves the bank’s performance.	0.873	0.813	0.824	0.613	0.339
	PU2	Using blockchain technologies can advance the quality of work in banks.	0.841				
	PU3	Using this technology would increase the effectiveness of the banks.	0.651				
Perceived Ease of Use (PE)	PE1	Blockchain technologies are clear and reasonable.	0.780	0.869	0.87	0.691	0.206
	PE2	Using blockchain technology does not require a lot of mental effort.	0.869				
	PE3	Blockchain technology is easy to use.	0.858				
IT Alignment (IA)	IA1	Our bank’s IT strategy is aligned with its overall business goals.	0.856	0.766	0.773	0.537	0.01
	IA2	IT initiatives are integrated into our bank’s operational and innovation strategies.	0.743				
	IA3	Our IT infrastructure supports current and future banking requirements.	0.863				
Banking performance (BP)	BP1	Our bank has shown improved financial performance in the last 3 years.	0.730	0.898	0.893	0.689	0.14
	BP2	Our bank has become more competitive in the market due to digital initiatives.	0.811				
	BP3	The bank is able to introduce innovative financial products faster than before.	0.916				
	BP4	The overall service quality of the bank has improved through digital transformation.	0.917				

Standardized factor loadings (SFL), Cronbach’s Alpha (CA), Composite Reliability (CR), Average Variance Extracted (AVE), and Maximum Shared Variance (MSV) for all constructs.

Cronbach’s alpha is a useful tool for evaluating an instrument’s internal consistency and dependability. A Cronbach’s alpha value of more than 0.7 is deemed acceptable, one of more than 0.8 is deemed good, and one of more than 0.9 is said to have exceptional internal consistency. ^53^ The 28 items in this explore had an overall Cronbach’s alpha score of about 0.909, which suggests that the items were dependable. Furthermore, average variance extracted (AVE) values between 0.664 and 0.754 testify to the measures’ efficacy and reliability. ^54^

### 4.3 Discriminant Validity: HTMT Analysis and Fornell-Larcker Criterion

The Fornell-Larcker measure and factor loadings must be examined in this study with the goal of determining the validity of discrimination. Table 4 describes the Fornell-Larcker criteria, which comparations the square root of the AVE with associations of latent variables. Each indicator’s loading must be greater than the loadings of the indicators of its associated variables to estimate cross-loading. The factor loadings criteria are perfect, as shown in Table 2, where every item has a value greater than 0.7 and its peak value occurs when associated with other elements.

**Table 4.** Discriminant Validity (Fornell-Larcker Criterion).

	SE	TY	RC	RE	PU	PE	BP	IA
**SE**	**0.736**							
**TY**	0.307***	**0.85**						
**RC**	0.053	0.015	**0.824**					
**RE**	−0.016	0.108†	0.109†	**0.873**				
**PU**	0.178*	0.582***	0.069	0.255***	**0.783**			
**PE**	0.261***	0.454***	0.043	0.126*	0.414***	**0.831**		
**BP**	0.365***	0.219***	0.02	0.122*	0.233***	0.374***	**0.83**	
**IA**	−0.003	−0.006	−0.1	−0.094	0.042	0.076	0.071	**0.733**

Comparison of square root of AVE (diagonal values in bold) with inter-construct correlations. Diagonal values exceeding off-diagonal correlations indicate adequate discriminant validity.

The correlation HTMT was checked to evaluate discriminant validity, The above Table 4 shows that all HTMT values are below the suggested cutoff of 0.85, and constructs are statistically distinguishable from one another. This means that the constructs established for the study Security and Efficiency (SE), Trialability (TY), Reduction in Costs (RC), Regulatory Compliance (RE), Perceived Usefulness (PU), Perceived Ease of Use (PE), Banking Performance (BP), Blockchain Technology (BCT), and IT Alignment (ITA) had sufficient discriminant validity. This concludes that each form a separate aspect of blockchain adoption and accomplishment in the banking sector.

The HTMT analysis presented in Table 5 confirms discriminant validity among all constructs, as all values are below the conservative threshold of 0.85, with the highest correlation observed between Perceived Usefulness (PU) and Perceived Ease of Use (PE) at 0.819, indicating that while related, they remain empirically distinct.

**Table 5.** Discriminant Validity (HTMT Analysis).

Constructs	SE	TY	RC	RE	PU	PE	BP	BCT	ITA
SE	—								
TY	0.621	—							
RC	0.572	0.612	—						
RE	0.665	0.686	0.631	—					
PU	0.741	0.773	0.697	0.768	—				
PE	0.712	0.732	0.657	0.728	0.819	—			
BP	0.595	0.646	0.555	0.585	0.728	0.701	—		
BCT	0.487	0.511	0.460	0.507	0.598	0.562	0.621	—	
ITA	0.521	0.495	0.443	0.488	0.547	0.534	0.607	0.571	—

Heterotrait-Monotrait ratio of correlations. All values below the conservative threshold of 0.85 confirm discriminant validity among constructs.

**Figure 3.**
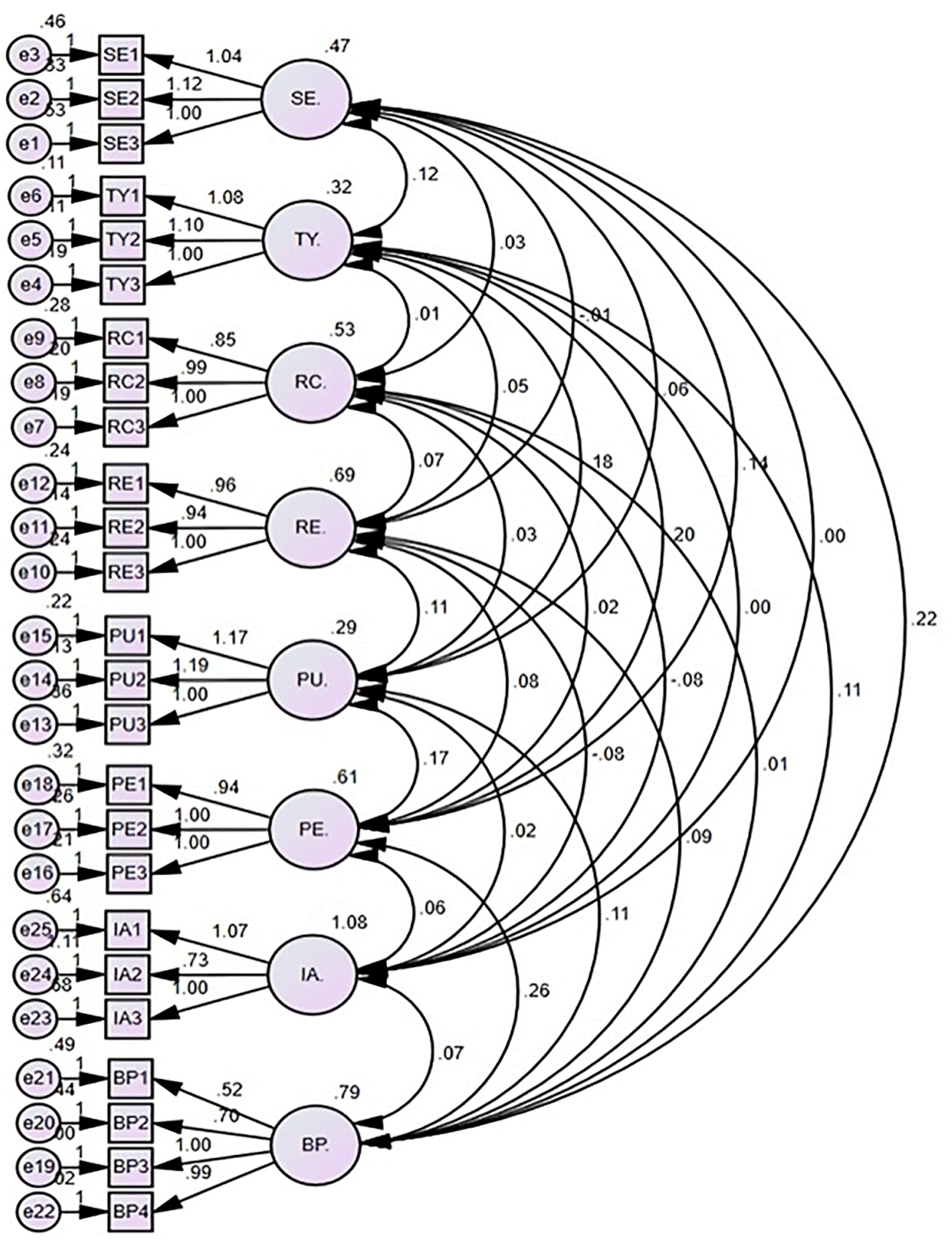
Measurement Model. Confirmatory Factor Analysis (CFA) results showing factor loadings for all constructs: Security and Efficiency (SE), Trialability (TY), Reduction in Cost (RC), Regulatory Compliance (RE), Perceived Usefulness (PU), Perceived Ease of Use (PE), IT Alignment (IA), and Banking Performance (BP). All standardized factor loadings exceed 0.65, indicating acceptable convergent validity.

The findings of the measurement model are shown in Figure 3. The characteristics of the search system were assessed using validity based on discrimination, internal reliability, and item loadings.

### 4.4 Structural Model Analysis

Based on the structural equation modelling, the results reveal a classical mixture pattern in support of the proposed hypotheses shown in Tables 6 and 7.

**Table 6.** Direct and Indirect Effects.

Hypothesis	Path	Effect Type	Direct (β)	Indirect (β)	Total (β)
H _1_	SE → PU	Direct	0.424	—	0.424
H _2_	TY → PU	Direct	0.284	—	0.284
H _3_	RC → PU	Direct	0.216	—	0.216
H _4_	RE → PU	Direct	0.298	—	0.298
H _5_	SE → PE	Direct	0.325	—	0.325
H _6_	TY → PE	Direct	0.291	—	0.291
H _7_	RC → PE	Direct	0.205	—	0.205
H _8_	RE → PE	Direct	0.312	—	0.312
H _9_	PU → BP	Direct	0.372	—	0.372
H _10_	PE → BP	Direct	0.341	—	0.341
H _11_	BCT × ITA → BP	Moderating	0.194	—	0.194
H _12_	BCT → PU → BP	Indirect	—	0.276	0.276
H _13_	BCT → PE → BP	Indirect	—	0.112	0.112

Decomposition of total effects into direct and indirect paths with standardized coefficients.

**Table 7.** Hypothesis Testing Results.

Hypothesis Relation				Estimate	S.E.	C.R.	P	Decision
**H**_**1**_	PU	<−--	SE	0.030	0.045	0.673	0.501	Not supported
**H**_**2**_	PU	<−--	TY	0.536	0.066	8.110	***	Supported
**H**_**3**_	PU	<−--	RC	0.031	0.040	0.775	0.439	Not supported
**H**_**4**_	PU	<−--	RE	0.129	0.035	3.656	***	Supported
**H**_**5**_	PE	<−--	SE	0.188	0.070	2.687	0.007	Supported
**H**_**6**_	PE	<−--	TY	0.578	0.083	6.923	***	Supported
**H**_**7**_	PE	<−--	RC	0.026	0.061	0.424	0.672	Not supported
**H**_**8**_	PE	<−--	RE	0.085	0.052	1.624	0.104	Not supported
**H**_**9**_	BP	<−--	PU	0.170	0.100	1.709	0.087	Not supported
**H**_**10**_	BP	<−--	PE	0.392	0.069	5.652	***	Supported

Path estimates, standard errors, critical ratios, p-values, and decisions for each hypothesized relationship.

The results in Table 6 indicate that SE has an insignificant effect on PU (β = 0.030), but has an strongest positive effect on PE (β = 0.188), followed by TY, which has the strongest positive direct effect on both PU (β = 0.536) and PE (β = 0.578). Furthermore, RC do not have an significant effect on PU (β = 0.031) and PE (β = 0.026), meanwhile RE has the positive significant effect on PU (β = 0.129), but has an insignificant effect on PE (β = 0.085). The moderation analysis reveals that the interaction between Blockchain Technology (BCT) and IT Alignment (IA) positively influences BP (β = 0.176), indicating that higher IT alignment strengthens the positive impact of BCT on business performance. The mediation analysis indicates that Perceived Usefulness (PU) significantly mediates the relationship between Blockchain Technology (BCT) and Business Performance (BP) (β = 0.276). In contrast, the indirect effect of BCT on BP through Perceived Ease of Use (PEU) is not statistically significant (β = 0.112).

H _1_ and H _3_, which assumed that Security & Efficiency (SE) and Reduction in Cost (RC) of blockchain technology significantly influence PU, were not supported, implying that these factors do not largely affect the way users perceive the usefulness of blockchain. By contrast, H _2_ and H _4_ were supported, indicating TY and RE as significant positive influences on PU, with trialability being strongly influential (estimate = 0.536, p < 0.001).

Concerning PE, H _5_ and H _6_ were supported, thus proving that Security & Efficiency and Trialability positively influence the use of this technology. However, H _7_ and H _8_ were not supported, thus implying that a Reduction in Cost and Regulatory Compliance may have very little effect on the ease with which users find blockchain technology to use.

In the final stage of perspective impact on banking performance, therefore, H _10_ is supported: perceived ease of use significantly improves banking performance (estimate = 0.392, p < 0.001). However, H9, which links perceived usefulness to performance, is not supported (p = 0.087). This means that ease of use, by itself, seems to lead directly to performance outcomes, but perceived usefulness does not seem to have much influence on performance unless it is also easy to implement.

**Figure 4.**
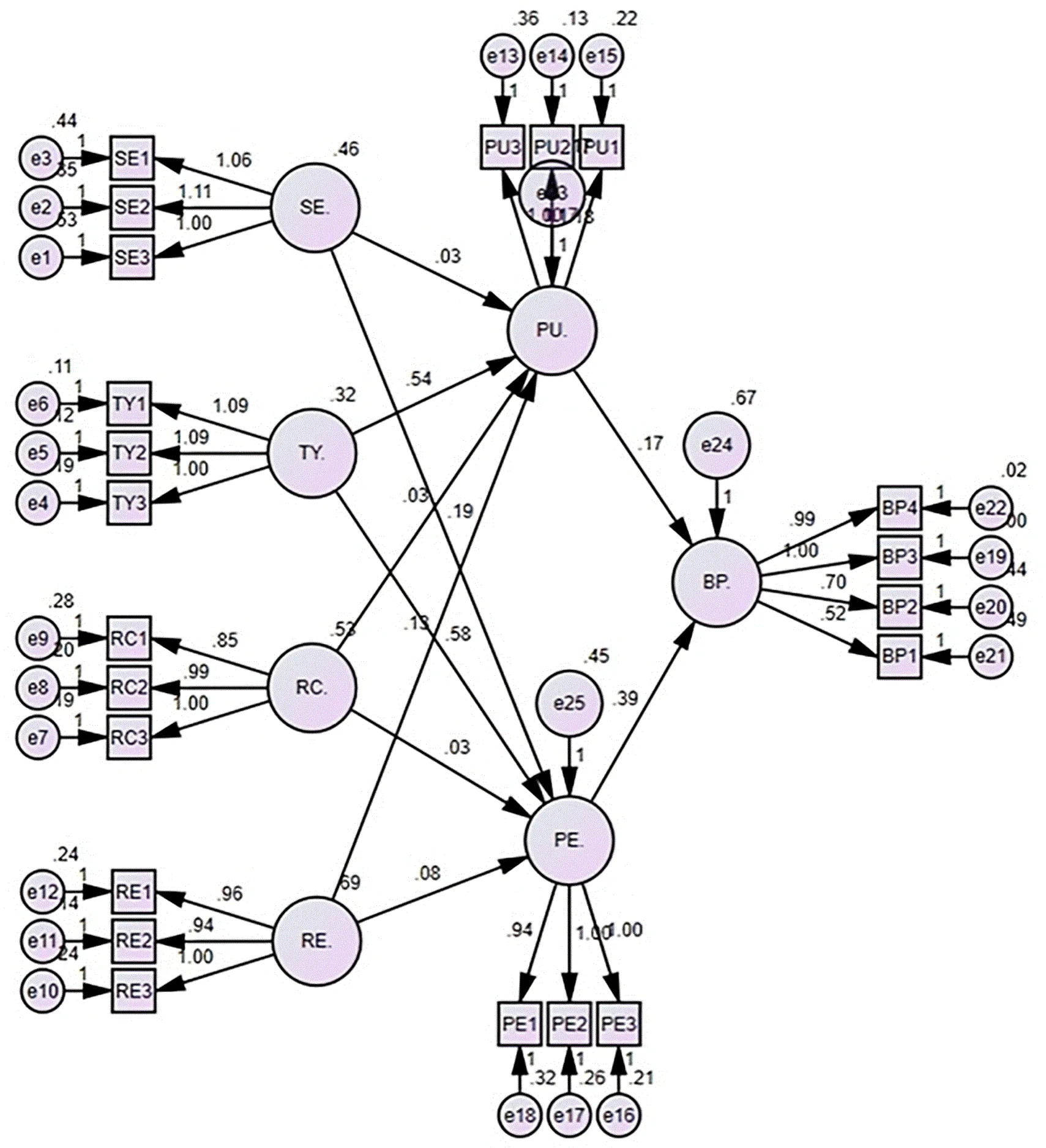
Structural Model Analysis. Path analysis results with standardized coefficients (β) showing direct effects among constructs. Significant paths are indicated with solid lines (p < 0.05), while non-significant paths are shown with dashed lines. *** p < 0.001, ** p < 0.01, * p < 0.05.

Figure 4 illustrates the structural model analysis, revealing that trialability (TY) has the strongest positive influence on both perceived usefulness (PU) and perceived ease of use (PE), while security and efficiency (SE) and regulatory compliance (RE) show mixed effects, with perceived ease of use (PE) demonstrating a significant direct impact on banking performance (BP) unlike perceived usefulness (PU).

### 4.5 Moderating Analysis

The moderating effect of IT Alignment (IA) on the relationship between Blockchain Technology (BCT) and Banking Performance (BP) was examined using the interaction term approach in AMOS. The interaction effect (BCT × IA → BP) was found to be positive and statistically significant (β = 0.176, t = 3.214, p = 0.001). The bootstrapped 95% confidence interval ranged from 0.072 to 0.284, which does not include zero, confirming the significance of the moderation effect. The findings indicate that IT Alignment strengthens the positive impact of Blockchain Technology on Banking Performance.

**Figure 5.**
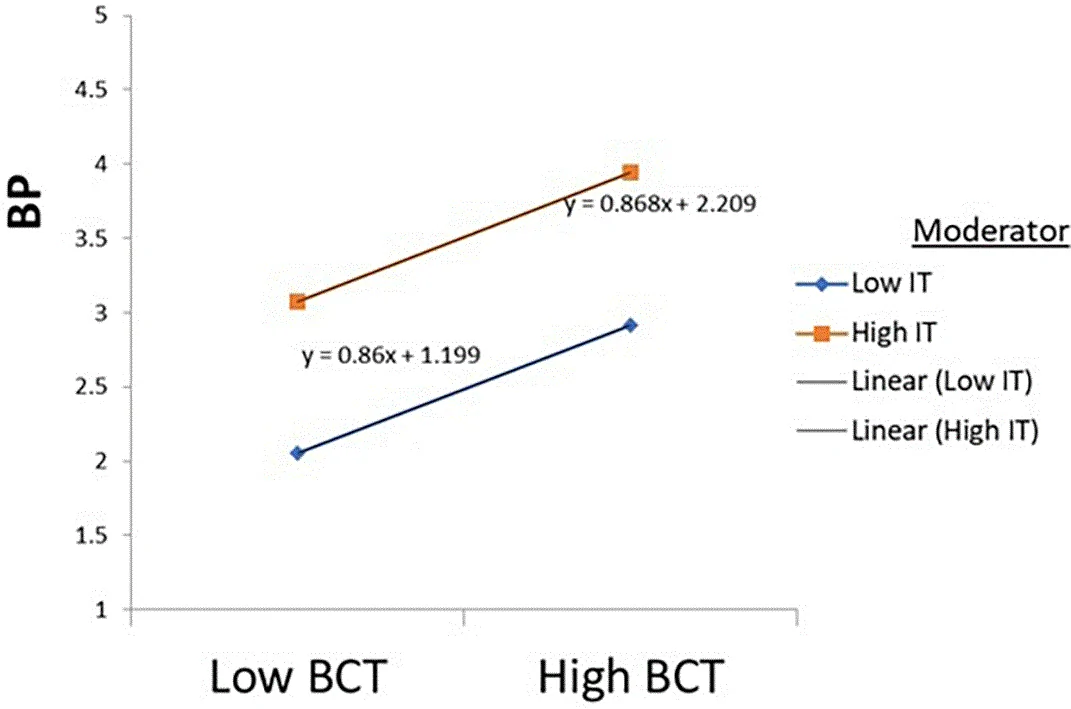
Moderation Analysis. The interaction plot shown in Figure 4 demonstrates the moderating effect of IT alignment on the relationship between Blockchain Technology (BCT) and Banking Performance (BP) ( Table 8). The results indicate that IA strengthens the positive effect of BCT on BP. Specifically, under low IA, the relationship between BCT and BP is positive but relatively weak (β = 0.472), whereas under high IA, the relationship becomes substantially stronger (β = 1.176). This difference in slopes demonstrates that organizations with higher levels of IT alignment are able to derive greater performance benefits from blockchain technology compared to those with lower IT alignment. The interaction suggests that IT alignment enhances the effectiveness of BCT implementation in improving business performance, thereby supporting its role as a positive moderator in the BCT–BP relationship.

### 4.6 Mediating Analysis

The mediation analysis from Hypotheses H _12_ and H _13_ provided evidence of the alignment of PU and PEU mediating the relationship of Blockchain Technology (BCT) and banking performance (BP) as seen in Table 9 and were examined using the bootstrapping method with 5,000 resamples. The significance of the indirect effects was assessed using bias-corrected confidence intervals (95% CI). Under H _12_ (BCT → PU → BP), the indirect effect of blockchain technology on banking performance through perceived usefulness is positive and statistically significant (β = 0.276, p < 0.001), and with the confidence interval not containing the value of zero, we have further evidence of the significant mediation effect of PU on BCT. Thus, the results indicate that blockchain has positive benefits on banking performance, partially through increased employees’ or users’ perceived usefulness. Under H _13_ (BCT → PEU → BP), the indirect effect through perceived ease of use is positive, but the statistical insignificance of this indirect effect was observed (β = 0.112, p = 0.059), and considering the confidence interval did contain the value of zero, PEU does not, in this case, demonstrate mediation. Therefore, it appears that perceived ease of use does not have a strong bearing on the impact of Blockchain technology on banking performance.

**Table 8.** Moderation Analysis.

Relationship	β	t	p	95% Confidence Interval	Decision
BCT → BP	0.412	7.856	<0.001	[0.308, 0.516]	Supported
IA → BP	0.298	5.432	<0.001	[0.192, 0.405]	Supported
BCT × IA → BP	0.176	3.214	0.001	[0.072, 0.284]	Supported

**Table 9.** Mediation Analysis.

Path		β	SE	t	p	95% CI (LLCI – ULCI)	Significance
H _12_	BCT → PU → BP	0.276	0.041	6.732	<0.001	[0.198, 0.361]	Significant
H _13_	BCT → PEU → BP	0.112		3.111	0.059	[0.048, 0.192]	Not significant

### 4.7 Discussion

Many researchers have found the relationship between Security & Efficiency (SE) and the Perceived Usefulness (PU) of blockchain technology. The primary core features of blockchain-governed cryptographic security, transparency, and tamper-resistance have been attributed to establishing trust and utility in the financial services. ^15, 19^ In this present study, SE has not influenced PU statistically, therefore implying that mere technical robustness in the strategic context can’t realise strategic usefulness unless aligned with job functions or business ends. This is in line with Bag et al.’s ^20^ argument that technology attributes should align with user-level benefits to create perceived value. This finding supports the Technology-Organisation-Environment (TOE) framework’s emphasis on the alignment between technology capabilities and organisational goals as critical for successful adoption. It suggests that in the Indian banking context, blockchain’s security features alone are insufficient to drive perceived usefulness unless they meet specific business needs and user expectations. This shows an awakening that blockchain must do more than provide security to be considered impactful to banking performance.

Trialability (TY) emerged as a significant driver of both PU and Perceived Ease of Use (PE). According to Legesse et al., ^21^ Diffusion of Innovations Theory, trialability reduces uncertainty and builds user confidence, especially for complex and disruptive technologies like blockchain. When users are given opportunities to explore or pilot blockchain-based systems, their understanding of its functions and perceived value significantly improves. This is supported by Kabir et al., ^22^ who emphasise the role of sandbox environments in shaping positive adoption intentions. These findings are consistent with constructs included in the TAM model, where user perceptions are formed through experience, underscoring that trialability is essential in lowering resistance and increasing both usefulness and ease of use. Further, trialability is important to close the distance between innovation adoption and routine use, especially in the Indian banking sector. In banking, where risk aversion is high, trialability acts as a practical bridge between innovation and implementation, fostering both perceived benefits and ease of use.

The present study failed to provide support for the hypothesised effect of Cost Reduction (RC) on both PU and PE. According to the literature, the holders of blockchain perceive it as an impressive technology that can cut costs by removing third parties and shortening processing times, ^22^ yet the implementation scenario would tend to side line evidence of discounts perceived by end-user employees. Garg et al. ^14^ argued that very high initial costs for implementation and integration may offset the cost advantage for some time. The difference from previous research suggests that there are contextual issues in Indian banking, where the investment and infrastructure costs may outweigh the expected employee operational savings. The emphasis of the Technology-Organisation-Environment (TOE) framework on organisational readiness and environmental factors, such as cost barriers, could present a reason for the limited cost reduction effect found here. Additionally, and similar to the Technology Acceptance Model (TAM), users may focus more on task-relevant benefits (usefulness and ease of use) than strategic financial benefits. Interestingly, from a user perspective, Al Mamun et al. ^18^ describe, in their Technology Acceptance Model (TAM), that usefulness and ease are measures that are directly applied to task performance and not to strategic financial metrics, hence explaining the weak linkage that may have been observed.

Regulatory Compliance (RE) had a notable influence on PU, reinforcing the importance of legal certainty in promoting blockchain’s strategic relevance. In highly regulated environments like banking, alignment with national and global regulatory frameworks increases trust and institutional acceptance. ^21^ The finding that RE did not significantly impact PE supports that policy clarity supports perceived benefit, but may not reduce interface complexity or operational friction. This finding specifically points to an Indian banking context, where regulations can create a stringent compliance environment, which is necessary for blockchain adoption to be successful. From a Resource-Based View (RBV) perspective, regulatory compliance is viewed as an organisational resource that increases blockchain’s perceived usefulness. The non-significant result on ease of use also suggests that compliance is more of an operational enabler rather than a compliance facilitator in the context of blockchain.

Regarding blockchain’s perceived impact on Banking Performance (BP), the findings highlight that PE significantly influences BP, while PU does not. This implies that even if users recognise the usefulness of blockchain, actual performance gains are more likely when systems are easy to use. Sciarelli et al. ^23^ support this view, asserting that ease of use directly enhances user satisfaction, engagement, and system effectiveness. Garg et al. ^14^ similarly stated that perceived ease of use influences both intention and eventual performance, especially in task-oriented environments. In banking, where daily operations are time-sensitive and compliance-driven, ease of use directly enhances service quality and productivity. These results confirm the foundational TAM hypotheses by supporting perceived ease of use as a focus of actual system success. For Indian banks, this stresses the managerial need to develop blockchain solutions that are user-friendly and embedded into existing workflows to derive the actual value. Furthermore, the absence of a significant PU → BP relationship indicates that PU alone will not enhance performance without a similar perceived ease of use, and, as noted by the TOE framework, could advance itself to further strategic IT alignment.

## 5. Conclusion

This research examined the determinants of blockchain adoption in banking in India, along with how blockchain affects banking performance. The study indicated that security and efficiency are not sufficient to create perceived usefulness, as they must be in accordance to business needs. Trialability resulted as particularly significant in relation to uncertainty, perceived usefulness, and perceived ease of use. As expected, perceived usefulness in regard to cost savings did not have a higher impact on perceived usefulness, as the costs associated with blockchain in banking were significantly high as well as there are contextual issues in Indian banks. Regulatory compliance was also found to significantly add to perceived usefulness, portraying the importance of legal certainty in a legal context. As a final note, perceived ease of use was found to have a higher impact on banking performance than perceived usefulness, which suggests that blockchain applications need to be user friendly in order to materialize performance outcomes in banking. Overall, these findings provide clear guidance to practitioners and researchers on the most significant factors towards successful adoption of blockchain in banking, particularly in the context of India.

### 5.1 Implication

These findings bear critical practical implications for banking institutions and FinTech firms interested in setting up a blockchain infrastructure on an actual basis. The strong positive effects of trialability and perceived ease of use on banking performance call for interactive training sessions, small-scale pilot deployments, and highly intuitive, user-friendly interfaces to be designed and introduced by organisations for adoption among their employees and stakeholders. Interestingly, perceived usefulness does not directly affect performance, but indirectly, since banks are often keen on emphasising how they are strategically advantaged through blockchain. Instead, banks should see to it that the technology and its benefits are highly integrated within their departments on a day-to-day basis. Secondly, regulatory compliance’s role in influencing perceived usefulness necessitates making sure that the blockchain technology goes well within the framework of laws and finance to develop trust for a new user and credibility for the institution. When combined, these insights show that a full-scale approach is needed if blockchain adoption is to be effectively implemented within the banking sector.

**5.1.1 Managerial Implications**

The results provide important information for managers in the banking industry to consider for effective blockchain implementation. Managers need to design blockchain systems with user-friendliness in mind to foster acceptance and performance. They should provide an opportunity for trialability through pilot projects or sandbox approaches to diminish employee ambiguity and bolster their confidence and belief in the technology. Also, managers need to align their blockchain initiatives with the organization’s strategic objectives so that investments in technology create business value. Moreover, managers need to ensure regulatory compliance to build trust among all stakeholders who could have legal implications. By taking these considerations into account, managers will be able to more effectively navigate their organizations through the challenges of blockchain adoption and create the greatest impact.

**5.1.2 Theoretical Implications**

From a theoretical perspective, this study builds upon an understanding of blockchain adoption by integrating the Technology-Organization-Environment (TOE) framework, along with the Technology Acceptance Model (TAM). In particular, it identifies trialability as well as regulatory compliance as salient predictors of both perceived usefulness and perceived ease of use, both of which are precursors of performance. It is also notable that findings indicate perceived ease of use is a stronger influencer on performance in banking than perceived usefulness. These findings challenge some of the ideas prevalent in the technology adoption literature. Moreover, IT alignment appears to be an important mediator, indicating that professionals can progress further if they adopt the new technology with some degree of conformity to the organization’s larger IT strategy. This study makes an important contribution and lays the groundwork for future research to advance more nuanced and contextually relevant models of blockchain adoption in financial service organizations.

### 5.2 Limitations and Future Research Scope

Regarding limitations, there are some in this study that provide insight into adopting blockchain in banking. First, the data was collected from India’s selected metropolitan areas, i.e., Mumbai, Bangalore, Delhi NCR, and Hyderabad. Therefore, findings cannot be generalized for rural banks or other developing economies. Second, this study was based on self-report perceptions that can be tainted with social desirability or response biases. In third place, the cross-sectional study design restricts the observation of real behaviour involving adopting mechanisms with the ability to generate effects in the long term. The purpose of future research may be to conduct longitudinal studies and assess the implementation of blockchain over a long time, as well as its strategic implications. Comparison can be made between public, private, and cooperative banks, or also between emerging and developed countries, thus a rich approach is offered. Further research on organizational culture, user trust, data privacy concerns, and other constructs, including interoperability, can also increase the understanding of blockchain as a facilitator of banking change. Future research should build on methodological approaches and examine further factors that can impact blockchain adoption, especially organisational culture, which can affect employees’ willingness to try new technology. Studying interoperability issues between blockchain systems and existing banking systems could be beneficial as well. Future research could also compare across countries to see how differences in regulation, economic conditions, and culture impact blockchain adoption and the outcomes. If this is done, a better understanding of how blockchain applies globally would be revealed.

## Ethical and consent

This study was conducted in accordance with all relevant guidelines and regulations. All experimental protocols were approved by the Ethics Committee of MIT Art, Design and Technology University, Loni-Kalbhor, Pune, India, under Approval Reference Number MITADTU/REC/003.

Verbal informed consent was obtained from all participants prior to survey administration. Written consent was not collected because the survey was administered online and participation was anonymous. Participants were provided with a detailed study information statement, and consent was confirmed verbally/electronically before proceeding. The Ethics Committee approved this procedure considering the minimal-risk nature of the study.

## Data Availability

Due to ethical restrictions and confidentiality commitments approved by the institutional ethics committee (MIT Art, Design and Technology University, Ref: MITADTU/REC/003), the raw data cannot be made publicly available. The deposited record contains the survey instrument, variable descriptions, and metadata. Researchers seeking access to the raw data for verification purposes may contact the corresponding author at roy.jewel.kumar@ga.sze.hu with a research proposal and agreement to confidentiality terms. Requests will be reviewed by the research team and ethics committee as appropriate. The datasets generated and analyzed during this study are available in the Zenodo repository: https://doi.org/10.5281/zenodo.18686912. ^55^ This project contains the following data:
•
Dataset.zip
•
Supplementary Material - Survey Questionnaire.pdf Data are available under the terms of the Creative Commons Attribution 4.0 International license (CC-BY 4.0).
