# Supplementary material for: Assessing factors driving blockchain technology in the performance of the banking sector using the structural equation modelling approach
Source: Open Res Eur. 2026 Aug 31;6:229. Originally published 2026 Jul 3. [Version 2] doi: 10.12688/openreseurope.23147.2 (PMC13601790; doi:10.12688/openreseurope.23147.2)
Supplement: Peer review history

# Reviewer report: Reviewer response for version 2

**DOI:** 10.21956/openreseurope.26982.r80100

**Published:** 2026-09-25

**Authors:** Sandy Francis Peris

**Affiliations:** 1 State University of Bangladesh, Dhaka, Bangladesh

Thanks for revised version

If applicable, is the statistical analysis and its interpretation appropriate?

Yes

Is the study design appropriate and is the work technically sound?

Yes

Is the work clearly and accurately presented and does it engage with the current literature?

Yes

Are the conclusions drawn adequately supported by the results?

Yes

Are sufficient details of methods and analysis provided to allow replication by others?

Yes

Are all the source data and materials underlying the results available?

Yes

Reviewer Expertise:

Sustainability, Techonology adaptation, Marketing, Entrepreneurship, SME

I confirm that I have read this submission and believe that I have an appropriate level of expertise to confirm that it is of an acceptable scientific standard.

# Reviewer report: Reviewer response for version 2

**DOI:** 10.21956/openreseurope.26982.r80099

**Published:** 2026-09-23

**Authors:** Abdullah Al Mamun

**Affiliations:** 1 Department of Business and Quantitative Studies, University of Naples Parthenope, Naples, Napoli, Italy

Accepted.

If applicable, is the statistical analysis and its interpretation appropriate?

Partly

Is the study design appropriate and is the work technically sound?

Partly

Is the work clearly and accurately presented and does it engage with the current literature?

Partly

Are the conclusions drawn adequately supported by the results?

Partly

Are sufficient details of methods and analysis provided to allow replication by others?

Partly

Are all the source data and materials underlying the results available?

Yes

Reviewer Expertise:

NA

I confirm that I have read this submission and believe that I have an appropriate level of expertise to confirm that it is of an acceptable scientific standard.

# Reviewer report: Reviewer response for version 1

**DOI:** 10.21956/openreseurope.25042.r76919

**Published:** 2026-07-23

**Authors:** Sandy Francis Peris

**Affiliations:** 1 State University of Bangladesh, Dhaka, Bangladesh

I am happy to contribute as a reviewer in this study. It is a good paper that studied the timely needed issue. 

 This manuscript applies an integrated TAM–TOE framework to examine blockchain technology adoption and its relationship with banking performance among 326 banking and FinTech professionals in India, with IT alignment tested as a moderator. The topic is timely and well within Open Research's scope, the sample size is adequate for SEM, and the measurement-model diagnostics (Cronbach's alpha, AVE, HTMT, common method bias checks) show methodological care. The hypothesis structure (H1–H13) is clearly laid out and easy to follow against the results.

 However, Section 4.4 contains a direct contradiction — it states that Security & Efficiency has "the strongest effect on PU" and then, one paragraph later, reports that the same path (H1) was *not* statistically significant. This needs to be resolved before the results can be taken at face value.

 I also have a concern about the paper's central claim regarding IT alignment. The moderation analysis reports nearly identical slopes for high vs. low IT alignment groups (0.86 vs. 0.868), which is not what a significant interaction effect looks like — that pattern is more consistent with a direct effect of IT alignment on performance than true moderation. The authors should report the interaction coefficient and its significance directly rather than inferring moderation from slope similarity.

 I hope these changes will upgrade the quality of the paper.

If applicable, is the statistical analysis and its interpretation appropriate?

Yes

Is the study design appropriate and is the work technically sound?

Yes

Is the work clearly and accurately presented and does it engage with the current literature?

Yes

Are the conclusions drawn adequately supported by the results?

Yes

Are sufficient details of methods and analysis provided to allow replication by others?

Yes

Are all the source data and materials underlying the results available?

Yes

Reviewer Expertise:

Sustainability, Techonology adaptation, Marketing, Entrepreneurship, SME

I confirm that I have read this submission and believe that I have an appropriate level of expertise to confirm that it is of an acceptable scientific standard.

# Reviewer report: Reviewer response for version 1

**DOI:** 10.21956/openreseurope.25042.r76917

**Published:** 2026-07-22

**Authors:** Abdullah Al Mamun

**Affiliations:** 1 Department of Business and Quantitative Studies, University of Naples Parthenope, Naples, Napoli, Italy

**Peer Review Report**

 Article: Assessing factors driving blockchain technology in the performance of the banking sector using the structural equation modelling approach

**Summary of the Article**

 This study surveys 326 banking and FinTech professionals across four Indian metros (Mumbai, Bangalore, Delhi NCR, Hyderabad) and uses Structural Equation Modelling (AMOS/SPSS) to test an integrated TAM-TOE model of blockchain adoption. Four antecedents — Security & Efficiency, Trialability, Reduction in Cost, and Regulatory Compliance — are hypothesised to shape Perceived Usefulness (PU) and Perceived Ease of Use (PE), which mediate the effect of blockchain technology on Banking Performance (BP), with IT Alignment proposed as a moderator of the technology-performance link. Reported findings: Trialability is the strongest driver of both PU and PE; PE (not PU) significantly predicts BP; Cost Reduction has no significant effect on either mediator; and IT Alignment moderates the BCT-BP relationship. The measurement model (reliability, convergent and discriminant validity) is well executed, but the structural results tables contain internal inconsistencies that need correcting before the substantive conclusions can be fully trusted.

**Answers to the Review Questions**

**Review Question**

**Answer**

 Is the work clearly and accurately presented and does it engage with the current literature?

**Partly**

 Is the study design appropriate and is the work technically sound?

**Partly**

 Are sufficient details of methods and analysis provided to allow replication by others?

**Partly**

 Are all the source data and materials underlying the results available?

**Yes**

 If applicable, is the statistical analysis and its interpretation appropriate?

**Partly**

 Are the conclusions drawn adequately supported by the results?

**Partly**

**Expanded Answers**

***Q1. Is the work clearly and accurately presented and does it engage with the current literature? — Partly***

 The literature review itself is organised well by theoretical lens (TAM, UTAUT, TOE, RBV), and Table 1's synthesis of prior empirical studies is genuinely useful. However, two things hold this back from a "Yes".

**Novelty and contribution are asserted rather than demonstrated. **The paper's own Table 1 already includes Garg et al. (2023) — SEM on blockchain capabilities and performance in the Indian banking sector — and Jena (2022) — extended UTAUT on blockchain adoption in Indian financial institutions and FinTech. These are precisely the "Indian banking plus blockchain plus SEM" studies the paper claims are missing from the literature. Section 2.2's stated gap is too generic to explain why these two studies do not already close it.

**Fix: **add an explicit paragraph (or comparison table) contrasting this study's model against Garg et al. and Jena specifically — for example, neither study tests IT Alignment as a moderator, and neither compares PU vs. PE as competing predictors of performance. Lead with those two points as the concrete contribution, rather than the generic "TOE+TAM integration" framing, since Legesse et al. (2024), already cited in this paper, has combined TOE and TAM before.

**Numerous minor factual and numerical inaccuracies in the text **also undermine "accurately presented": the text claims AVE ranges "between 0.664 and 0.754," but Table 3 shows 0.537-0.762; the text claims "28 items" were measured, but Table 2/3 list 25; RQ2 asks whether IT Alignment "mediates" the BCT-performance link while H11 and Section 2.4.4 call it a moderator. This mediator/moderator inconsistency should be resolved throughout, including the abstract. A language-editing pass would also help (recurring grammatical slips such as "will serves," "despite of," "stuffs" for items, "dignified" for rated, "touches" for influences).

***Q2. Is the study design appropriate and is the work technically sound? — Partly***

 The overall design — a cross-sectional TAM/TOE survey analysed via CFA and structural SEM — is appropriate for the stated research questions, and the sample size (326, purposive sampling across bank types) is adequate for the model's complexity. Two technical gaps prevent a clean "Yes".

**The moderation test (H11) is never formally specified. **The paper does not state which method was used to test IT Alignment as a moderator (product-indicator SEM, latent moderated structural equations, a PROCESS-style regression, or a simple median-split comparison). The interaction plot in Figure 5 shows two regression lines with almost identical slopes (0.86 vs. 0.868) but different intercepts — a pattern that normally indicates a main effect of the moderator on the outcome, not an interaction/moderation effect on the BCT-to-BP slope. As written, the "IT Alignment moderates baseline performance rather than the slope" conclusion is a non-standard use of "moderation" and should either be reframed as a main effect or supported with a formal interaction-term significance test.

**The "Blockchain Technology" (BCT) construct **is used in H11 and in the HTMT table (Table 5) without ever being operationalised with its own items in Table 2/3. It is unclear whether BCT is a formative second-order construct built from SE/TY/RC/RE or measured independently. This needs to be specified for the model to be technically transparent.

***Q3. Are sufficient details of methods and analysis provided to allow replication by others? — Partly***

 The survey instrument, constructs, sampling frame, and software (SPSS 20 for EFA, AMOS 26.0 for CFA) are reported. However:

- EFA is mentioned but no EFA output is shown — no KMO/Bartlett's test, no eigenvalues/scree plot, no rotated factor loadings. Either report this output or clarify why only CFA results are presented.
- The moderation-analysis method is not described (see Q2) — a replicator could not reproduce Figure 5 or the H11 estimate from the Methods section as written.
- Bootstrap parameters for the mediation analysis (number of resamples, bias-corrected vs. percentile CIs) are not stated anywhere, which is needed to reproduce Table 8.

***Q4. Are all the source data and materials underlying the results available? — Yes***

 A Zenodo DOI (10.5281/zenodo.18686912) is provided, along with the survey instrument and metadata, and the data-availability statement explains the confidentiality basis for withholding raw responses while offering a contact route for verification. I was not able to independently confirm the record resolves, but the reporting practice itself (DOI, instrument, and access process) meets the expectation for this criterion.

***Q5. If applicable, is the statistical analysis and its interpretation appropriate? — Partly***

 This is the area needing the most attention, and where I would draw the clearest line for what must be fixed.

**Table 6 and Table 7 report materially different coefficients for the same ten hypotheses, **and Table 6 does not match Figure 4's path diagram either (which agrees with Table 7). For example, H1 (SE to PU) is 0.424 in Table 6 but 0.030, not significant, in Table 7; H9 (PU to BP) is 0.372 in Table 6 but 0.170, not significant, in Table 7. This is not a rounding discrepancy — it is a wholesale mismatch across every path, and it means a reader cannot currently determine the actual model output.

**Table 8 (mediation results) reports the coefficient, standard error, t-value, p-value, and confidence interval as the identical single number **in each row (0.276 for H12, 0.112 for H13) — statistically impossible as printed, and it needs to be regenerated with genuine bootstrap output (distinct SE, t or z value, p, and asymmetric CI bounds).

**The text and Table 8 contradict each other on H13: **Section 4.6 says the indirect effect is not significant (p = 0.059, CI includes zero — mediation not supported), while Table 8 lists it as "Significant" with a different p-value (0.112).

**H11's significance is never reported **with a standard error, critical ratio, or p-value anywhere in the paper.

***Q6. Are the conclusions drawn adequately supported by the results? — Partly***

 Several conclusions (Trialability as the strongest driver of PU/PE; PE outperforming PU in predicting BP; Cost Reduction's limited effect) are consistent with Table 7 and Figure 4, and would be reasonably well supported if Table 6 and Table 8 are corrected to match. But as the paper stands, a conclusion such as "IT Alignment plays a significant moderating role" (stated in the abstract) currently rests on a single unlabelled point estimate with no significance test, and the PU-to-BP interpretation shifts depending on which of Table 6 or Table 7 a reader relies on. The conclusions cannot be judged as fully supported until the underlying tables agree with each other.

**Points That Must Be Addressed for the Article to Be Scientifically Sound**

1. Reconcile Table 6, Table 7, and Figure 4 so all three report the same coefficients for H1 through H10, with non-significant paths clearly flagged.
2. Regenerate Table 8 with genuine, non-identical bootstrap statistics (standard error, t or z value, p-value, distinct confidence interval bounds), and align it with the narrative in Section 4.6.
3. Report a formal significance test (estimate, standard error, critical ratio, p-value) for H11, and specify the moderation-testing method used.
4. Clarify or reframe the moderation interpretation in light of the near-identical slopes in Figure 5.
5. Specify how "Blockchain Technology" was operationalised as a construct for H11 and Table 5.
6. Correct the AVE range, item count, and mediator/moderator terminology inconsistencies between text and tables.
7. Sharpen the novelty claim with an explicit contrast against Garg et al. (2023) and Jena (2022), the two closest prior Indian-banking blockchain-SEM studies already cited in the paper.

If applicable, is the statistical analysis and its interpretation appropriate?

Partly

Is the study design appropriate and is the work technically sound?

Partly

Is the work clearly and accurately presented and does it engage with the current literature?

Partly

Are the conclusions drawn adequately supported by the results?

Partly

Are sufficient details of methods and analysis provided to allow replication by others?

Partly

Are all the source data and materials underlying the results available?

Yes

Reviewer Expertise:

NA

I confirm that I have read this submission and believe that I have an appropriate level of expertise to confirm that it is of an acceptable scientific standard, however I have significant reservations, as outlined above.

# Response

**Published:** 2026-08-06

**Authors:** Jewel Kumar Roy

Dear Sir We appreciate the reviewer for reviewing our paper and providing valuable comments. We hope the manuscript after careful revisions meets your high standards. Below we provide the point-by-point responses. All the modifications have been highlighted in blue. **Peer Review Report**

 Article: Assessing factors driving blockchain technology in the performance of the banking sector using the structural equation modelling approach

**Summary of the Article**

 This study surveys 326 banking and FinTech professionals across four Indian metros (Mumbai, Bangalore, Delhi NCR, Hyderabad) and uses Structural Equation Modelling (AMOS/SPSS) to test an integrated TAM-TOE model of blockchain adoption. Four antecedents — Security & Efficiency, Trialability, Reduction in Cost, and Regulatory Compliance — are hypothesised to shape Perceived Usefulness (PU) and Perceived Ease of Use (PE), which mediate the effect of blockchain technology on Banking Performance (BP), with IT Alignment proposed as a moderator of the technology-performance link. Reported findings: Trialability is the strongest driver of both PU and PE; PE (not PU) significantly predicts BP; Cost Reduction has no significant effect on either mediator; and IT Alignment moderates the BCT-BP relationship. The measurement model (reliability, convergent and discriminant validity) is well executed, but the structural results tables contain internal inconsistencies that need correcting before the substantive conclusions can be fully trusted. **Response: **Thank you for the positive feedback. As suggested, the internal inconsistencies identified in the structural results tables have been corrected. The revised structural model results have been updated in Section 4.4: Structural Model Analysis.

**Review Question** Is the work clearly and accurately presented and does it engage with the current literature?

**Partly** **Response: **As per the suggestion, the revised manuscript includes relevant recent studies and strengthens the connection with existing literature. The literature review (Section 2), research gap (Section 2.2), and contributions of the study (Section 1.1) have been explained more clearly.

 Is the study design appropriate and is the work technically sound?

**Partly** **Response: **As per the suggestion, the revised manuscript explains the research methods (Section 3), data analysis, and validation procedures (Section 4) more clearly. The selected methods are appropriate and support the reliability of the study findings.

 Are sufficient details of methods and analysis provided to allow replication by others?

**Partly** **Response: **As per the suggestion, the revised manuscript provides details about the research methods and analysis procedures. The data collection process and instrument (Section 3.1), measurement of constructs (Section 3.2), and data analysis procedures (Section 4) have been explained clearly. These improvements enhance the clarity and reliability of the study.

 Are all the source data and materials underlying the results available?

**Yes** **Response:** Thank you for your positive evaluation.

 If applicable, is the statistical analysis and its interpretation appropriate?

**Partly** **Response:** The manuscript has been revised to improve the explanation of the statistical methods and the interpretation of the results.

 Are the conclusions drawn adequately supported by the results?

**Partly** **Response: **The manuscript has been revised. The results are linked with the conclusions, and additional explanations have been added to improve the clarity and support of the findings.

**Expanded Answers**

***Q1. Is the work clearly and accurately presented and does it engage with the current literature? — Partly***

 The literature review itself is organised well by theoretical lens (TAM, UTAUT, TOE, RBV), and Table 1's synthesis of prior empirical studies is genuinely useful. However, two things hold this back from a "Yes". **Novelty and contribution are asserted rather than demonstrated. **The paper's own Table 1 already includes Garg et al. (2023) — SEM on blockchain capabilities and performance in the Indian banking sector — and Jena (2022) — extended UTAUT on blockchain adoption in Indian financial institutions and FinTech. These are precisely the "Indian banking plus blockchain plus SEM" studies the paper claims are missing from the literature. Section 2.2's stated gap is too generic to explain why these two studies do not already close it.

**Fix: **add an explicit paragraph (or comparison table) contrasting this study's model against Garg et al. and Jena specifically — for example, neither study tests IT Alignment as a moderator, and neither compares PU vs. PE as competing predictors of performance. Lead with those two points as the concrete contribution, rather than the generic "TOE+TAM integration" framing, since Legesse et al. (2024), already cited in this paper, has combined TOE and TAM before. **Response: **As per the suggestion, Section 1.1 has been updated with the research contribution. Table 1 has been revised for a clearer comparison with previous studies. Section 2.2 has been revised with a clear research gap by comparing the present study with Garg et al. (2023) and Jena (2022) and highlighting IT Alignment as a moderating variable and the comparison of Perceived Usefulness (PU) and Performance Expectancy (PE).

**Numerous minor factual and numerical inaccuracies in the text **also undermine "accurately presented": the text claims AVE ranges "between 0.664 and 0.754," but Table 3 shows 0.537-0.762; the text claims "28 items" were measured, but Table 2/3 list 25; RQ2 asks whether IT Alignment "mediates" the BCT-performance link while H11 and Section 2.4.4 call it a moderator. This mediator/moderator inconsistency should be resolved throughout, including the abstract. A language-editing pass would also help (recurring grammatical slips such as "will serves," "despite of," "stuffs" for items, "dignified" for rated, "touches" for influences). **Response: **As per the suggestion, the identified errors and inconsistencies have been corrected throughout the manuscript. The AVE values, the number of measurement items, and the role of IT Alignment have been updated to ensure consistency in the abstract, research questions, hypotheses, tables, and the main text. The manuscript has been proofread, and all grammatical and typographical errors have been corrected.

***Q2. Is the study design appropriate and is the work technically sound? — Partly***

 The overall design — a cross-sectional TAM/TOE survey analysed via CFA and structural SEM — is appropriate for the stated research questions, and the sample size (326, purposive sampling across bank types) is adequate for the model's complexity. Two technical gaps prevent a clean "Yes".

**The moderation test (H11) is never formally specified. **The paper does not state which method was used to test IT Alignment as a moderator (product-indicator SEM, latent moderated structural equations, a PROCESS-style regression, or a simple median-split comparison). The interaction plot in Figure 5 shows two regression lines with almost identical slopes (0.86 vs. 0.868) but different intercepts — a pattern that normally indicates a main effect of the moderator on the outcome, not an interaction/moderation effect on the BCT-to-BP slope. As written, the "IT Alignment moderates baseline performance rather than the slope" conclusion is a non-standard use of "moderation" and should either be reframed as a main effect or supported with a formal interaction-term significance test. **Response: **As per the comment, the moderation analysis has been clarified in **Section 4.5**. The method used to test the moderating effect of IT Alignment and the interpretation of the results have been revised and presented clearly.

**The "Blockchain Technology" (BCT) construct **is used in H11 and in the HTMT table (Table 5) without ever being operationalised with its own items in Table 2/3. It is unclear whether BCT is a formative second-order construct built from SE/TY/RC/RE or measured independently. This needs to be specified for the model to be technically transparent. **Response: **As suggested, Blockchain Technology (BCT) is modeled as a **second-order reflective construct** comprising four first-order dimensions: Security and Efficiency (SE), Trialability (TY), Reduction in Cost (RC), and Regulatory Compliance (RE). Since BCT is operationalized through these dimensions, it does not have separate measurement items. This clarification has been added to the **Second-Order Construct Specification** subsection (3.3.1) of the Methodology

***Q3. Are sufficient details of methods and analysis provided to allow replication by others? — Partly***

 The survey instrument, constructs, sampling frame, and software (SPSS 20 for EFA, AMOS 26.0 for CFA) are reported. However:

- EFA is mentioned but no EFA output is shown — no KMO/Bartlett's test, no eigenvalues/scree plot, no rotated factor loadings. Either report this output or clarify why only CFA results are presented.

**Response: **As per the comment, The EFA results have been added in Section 4.2: Measurement Model Analysis. The EFA and CFA results have been explained, and the KMO/Bartlett's test, eigenvalues, scree plot, and factor loadings have been included in Table 3: Measurement Model.  

- The moderation-analysis method is not described (see Q2) — a replicator could not reproduce Figure 5 or the H11 estimate from the Methods section as written.

**Response: **As per the suggestion, the moderation analysis has been updated and explained in Section 4.5.  

- Bootstrap parameters for the mediation analysis (number of resamples, bias-corrected vs. percentile CIs) are not stated anywhere, which is needed to reproduce Table 8.

**Response: **As per the comment, the bootstrap parameters for the mediation analysis have been added in **Section 4.6** **.** ***Q4. Are all the source data and materials underlying the results available? — Yes***

 A Zenodo DOI (10.5281/zenodo.18686912) is provided, along with the survey instrument and metadata, and the data-availability statement explains the confidentiality basis for withholding raw responses while offering a contact route for verification. I was not able to independently confirm the record resolves, but the reporting practice itself (DOI, instrument, and access process) meets the expectation for this criterion. **Response: **Thank you for your positive feedback.

***Q5. If applicable, is the statistical analysis and its interpretation appropriate? — Partly***

 This is the area needing the most attention, and where I would draw the clearest line for what must be fixed.

**Table 6 and Table 7 report materially different coefficients for the same ten hypotheses, **and Table 6 does not match Figure 4's path diagram either (which agrees with Table 7). For example, H1 (SE to PU) is 0.424 in Table 6 but 0.030, not significant, in Table 7; H9 (PU to BP) is 0.372 in Table 6 but 0.170, not significant, in Table 7. This is not a rounding discrepancy — it is a wholesale mismatch across every path, and it means a reader cannot currently determine the actual model output. **Response: **As per the reviewer’s comment, the inconsistencies between Table 6, Table 7, and Figure 3 have been checked and corrected. The revised tables and path model now present consistent results for all hypotheses.

**Table 8 (mediation results) reports the coefficient, standard error, t-value, p-value, and confidence interval as the identical single number **in each row (0.276 for H12, 0.112 for H13) — statistically impossible as printed, and it needs to be regenerated with genuine bootstrap output (distinct SE, t or z value, p, and asymmetric CI bounds). **Response: **As per the suggestion, the mediation results table has been checked and corrected. The revised mediation analysis for Hypotheses H12 and H13 is presented in Table 9, showing the updated bootstrap results and the mediating effects of PU and PEU on the relationship between Blockchain Technology (BCT) and Banking Performance (BP).

**The text and Table 8 contradict each other on H13:** Section 4.6 says the indirect effect is not significant (p = 0.059, CI includes zero — mediation not supported), while Table 8 lists it as "Significant" with a different p-value (0.112).

**H11's significance is never reported** with a standard error, critical ratio, or p-value anywhere in the paper. **Response: **As suggested, the inconsistencies related to H13 and H11 have been checked and corrected. The revised mediation results are presented in Table 9: Mediation Analysis.

***Q6. Are the conclusions drawn adequately supported by the results? — Partly***

 Several conclusions (Trialability as the strongest driver of PU/PE; PE outperforming PU in predicting BP; Cost Reduction's limited effect) are consistent with Table 7 and Figure 4, and would be reasonably well supported if Table 6 and Table 8 are corrected to match. But as the paper stands, a conclusion such as "IT Alignment plays a significant moderating role" (stated in the abstract) currently rests on a single unlabelled point estimate with no significance test, and the PU-to-BP interpretation shifts depending on which of Table 6 or Table 7 a reader relies on. The conclusions cannot be judged as fully supported until the underlying tables agree with each other. **Response: **As per the suggestion, the results, tables, and conclusions have been reviewed and revised. The inconsistencies between Table 6, Table 7, Table 8, and Figure 3 have been corrected, and the findings related to Trialability, PU, PE, Cost Reduction, and IT Alignment have been updated based on the revised analysis. The abstract and conclusion sections have been updated based on the revised results.

**Points That Must Be Addressed for the Article to Be Scientifically Sound**

1. Reconcile Table 6, Table 7, and Figure 4 so all three report the same coefficients for H1 through H10, with non-significant paths clearly flagged.

**Response: **As per the comment, the model results were re-run, checked, and corrected. Table 6, Table 7, and Figure 3 have been updated to ensure that the reported coefficients and significance levels are consistent for all hypotheses.  

1. Regenerate Table 8 with genuine, non-identical bootstrap statistics (standard error, t or z value, p-value, distinct confidence interval bounds), and align it with the narrative in Section 4.6.

**Response: **As per the comment, the table has been regenerated as recommended. Please note that the correct table number is Table 9, not Table 8. The results in Table 9 were examined using the bootstrapping method, and the values have been updated. The results are also consistent with the explanation provided in Section 4.6.  

1. Report a formal significance test (estimate, standard error, critical ratio, p-value) for H11, and specify the moderation-testing method used.

**Response: **As recommended, the moderating analysis has been updated in Section 4.5, and the formal significance test results for H11, including the estimate, standard error, critical ratio, and p-value, have been reported. The moderation-testing method used is also specified in Table 8: Moderation Analysis.  

1. Clarify or reframe the moderation interpretation in light of the near-identical slopes in Figure 5.

**Response: **As per the comment, the moderation interpretation has been revised and clarified based on the observed slopes. Please note that the correct figure reference is Figure 4, not Figure 5. The interpretation has been updated accordingly to ensure consistency with Figure 4.  

1. Specify how "Blockchain Technology" was operationalised as a construct for H11 and Table 5.

**Response: **As suggested, the operationalisation of the Blockchain Technology construct has been clarified in Section 3.3.1: Second-Order Construct Specification.  

1. Correct the AVE range, item count, and mediator/moderator terminology inconsistencies between text and tables.

**Response: **As per the comment, the manuscript has been checked and revised. The inconsistencies related to the AVE range, item count, and mediator/moderator terminology between the text and the tables have been corrected to ensure consistency throughout the manuscript.  

1. Sharpen the novelty claim with an explicit contrast against Garg et al. (2023) and Jena (2022), the two closest prior Indian-banking blockchain-SEM studies already cited in the paper.

**Response: **As per the comment, the novelty of the study has been clarified in Section 1.1 (Research Contribution) by adding an explicit comparison with Garg et al. (2023) and Jena (2022). Table 1 and Section 2.2 have also been revised to clearly highlight the differences between the previous studies and the present study. **Is the work clearly and accurately presented and does it engage with the current literature?** Partly **Response:** As per the suggestion, the revised manuscript includes relevant recent studies and strengthens the connection with existing literature. The literature review (Section 2), research gap (Section 2.2), and contributions of the study (Section 1.1) have been explained more clearly. **Is the study design appropriate and is the work technically sound?** Partly **Response: **As per the suggestion, the revised manuscript explains the research methods (Section 3), data analysis, and validation procedures (Section 4) more clearly. The selected methods are appropriate and support the reliability of the study findings. **Are sufficient details of methods and analysis provided to allow replication by others?** Partly **Response: **As per the suggestion, the revised manuscript provides details about the research methods and analysis procedures. The data collection process and instrument (Section 3.1), measurement of constructs (Section 3.2), and data analysis procedures (Section 4) have been explained clearly. These improvements enhance the clarity and reliability of the study. **Are all the source data and materials underlying the results available?** Yes **Response: **Thank you for your positive feedback. **If applicable, is the statistical analysis and its interpretation appropriate?** Partly **Response: **The manuscript has been revised to improve the explanation of the statistical methods and the interpretation of the results. **Are the conclusions drawn adequately supported by the results?** Partly **Response: **The manuscript has been revised. The results are linked with the conclusions, and additional explanations have been added to improve the clarity and support of the findings.
